# Thiopurine Enhanced ALL Maintenance (TEAM): study protocol for a randomized study to evaluate the improvement in disease-free survival by adding very low dose 6-thioguanine to 6-mercaptopurine/methotrexate-based maintenance therapy in pediatric and adult patients (0–45 years) with newly diagnosed B-cell precursor or T-cell acute lymphoblastic leukemia treated according to the intermediate risk-high group of the ALLTogether1 protocol

**DOI:** 10.1186/s12885-022-09522-3

**Published:** 2022-05-02

**Authors:** Linea Natalie Toksvang, Bodil Als-Nielsen, Christopher Bacon, Ruta Bertasiute, Ximo Duarte, Gabriele Escherich, Elín Anna Helgadottir, Inga Rinvoll Johannsdottir, Ólafur G. Jónsson, Piotr Kozlowski, Cecilia Langenskjöld, Kristi Lepik, Riitta Niinimäki, Ulrik Malthe Overgaard, Mari Punab, Riikka Räty, Heidi Segers, Inge van der Sluis, Owen Patrick Smith, Marion Strullu, Goda Vaitkevičienė, Hilde Skuterud Wik, Mats Heyman, Kjeld Schmiegelow

**Affiliations:** 1 Department of Pediatrics and Adolescent Medicine, Copenhagen University Hospital Rigshospitalet, Blegdamsvej 9, 2100 Copenhagen, Denmark; 2 St. James Hospital, Dublin, Ireland; 3 Vilnius University Hospital Santariskiu Klinikos, Vilnius, Lithuania; 4 Instituto Português de Oncologia Lisboa Francisco Gentil Departamento de Pediatria, Lisbon, Portugal; 5 University Medical Center Hamburg-Eppendorf, Hamburg, Germany; 6 Landspitali University Hospital, Reykjavik, Iceland; 7 Oslo University Hospital, Oslo, Norway; 8 Örebro University Hospital, Örebro, Sweden; 9 Drottning Silvias Barnsjukhus, Gothenburg, Sweden; 10 Tallinn Children’s Hospital, Tallinn, Estonia; 11 Oulu University Hospital and PEDEGRO Research Unit, University of Oulu, Oulu, Finland; 12 Copenhagen University Hospital Rigshospitalet, Copenhagen, Denmark; 13 Tartu University Hospital, Tartu, Estonia; 14 Helsinki University Central Hospital, Helsinki, Finland; 15 Leuvens Kanker Instituut (LKI), KU Leuven – UZ Leuven, Leuven, Belgium; 16 Princess Maxima Center for Pediatric Oncology, Utrecht, Netherlands; 17 Our Lady’s Children Hospital, Dublin, Ireland; 18 Université de Paris, hôpital universitaire Robert-Debré (APHP), Paris, France; 19 Center for Pediatric Oncology and Hematology, Vilnius University, Vilnius, Lithuania; 20 Karolinska Institutet, Stockholm, Sweden; 21 Astrid Lindgren Children’s Hospital, Karolinska University Hospital, Stockholm, Sweden; 22 University of Copenhagen, Copenhagen, Denmark

**Keywords:** Acute lymphoblastic leukemia, Maintenance, Thiopurines, 6-mercaptopurine, Methotrexate, 6-thioguanine, DNA-TG

## Abstract

**Background:**

A critical challenge in current acute lymphoblastic leukemia (ALL) therapy is treatment intensification in order to reduce the relapse rate in the subset of patients at the highest risk of relapse. The year-long maintenance phase is essential in relapse prevention. The Thiopurine Enhanced ALL Maintenance (TEAM) trial investigates a novel strategy for ALL maintenance.

**Methods:**

TEAM is a randomized phase 3 sub-protocol to the ALLTogether1 trial, which includes patients 0–45 years of age with newly diagnosed B-cell precursor or T-cell ALL, and stratified to the intermediate risk-high (IR-high) group, in 13 European countries. In the TEAM trial, the traditional methotrexate (MTX)/6-mercaptopurine (6MP) maintenance backbone (control arm) is supplemented with low dose (2.5–12.5 mg/m^2^/day) oral 6-thioguanine (6TG) (experimental arm), while the starting dose of 6MP is reduced from 75 to 50 mg/m^2^/day. A total of 778 patients will be included in TEAM during ~ 5 years. The study will close when the last included patient has been followed for 5 years from the end of induction therapy. The primary objective of the study is to significantly improve the disease-free survival (DFS) of IR-high ALL patients by adding 6TG to 6MP/MTX-based maintenance therapy. TEAM has 80% power to detect a 7% increase in 5-year DFS through a 50% reduction in relapse rate. DFS will be evaluated by intention-to-treat analysis. In addition to reducing relapse, TEAM may also reduce hepatotoxicity and hypoglycemia caused by high levels of methylated 6MP metabolites. Methotrexate/6MP metabolites will be monitored and low levels will be reported back to clinicians to identify potentially non-adherent patients.

**Discussion:**

TEAM provides a novel strategy for maintenance therapy in ALL with the potential of improving DFS through reducing relapse rate. Potential risk factors that have been considered include hepatic sinusoidal obstruction syndrome/nodular regenerative hyperplasia, second cancer, infection, and osteonecrosis. Metabolite monitoring can potentially increase treatment adherence in both treatment arms.

**Trial registration:**

EudraCT, 2018–001795-38. Registered 2020-05-15,

Clinicaltrials.gov, NCT04307576. Registered 2020-03-13, *https://clinicaltrials.gov/ct2/show/NCT04307576*

**Supplementary Information:**

The online version contains supplementary material available at 10.1186/s12885-022-09522-3.

## Background

The likelihood of cure of acute lymphoblastic leukemia (ALL) correlates with immunophenotype (B-cell precursor (BCP) or T-lineage), the mutational landscape of the leukemic cells, tumor burden (incl. white blood cell count (WBC) at diagnosis), central nervous system (CNS) involvement, and early response to therapy as measured by measurable residual disease (MRD) in the bone-marrow during the first months of therapy [1].

In the ALLTogether1 protocol, these features stratify patients to standard risk, intermediate risk (IR)-low, IR-high, and high risk groups. Patients from the legacy protocols, who retrospectively were re-stratified according to the ALLTogether1 IR-high criteria had a significantly worse outcome than patients retrospectively re-stratified as IR-low patients (5-year event-free survival (EFS): 82% vs. 94%), and the majority (> 60%) of all anticipated relapses in the ALLTogether1 protocol are expected to occur in the IR-high group. Only 50–60% of these relapses can be expected to be successfully salvaged by 2nd line therapy [2, 3]. Adult patients (18–45 years) have an even worse prognosis after relapse (overall survival (OS) 10–20%) [4–6].

Maintenance therapy is the last and longest phase of antileukemic chemotherapy, comprised of the cornerstones daily oral 6-mercaptopurine (6MP) (50–75 mg/m^2^) and weekly oral methotrexate (MTX) (usually 20 mg/m^2^), and plays a key role in the cure of ALL. Thus, shorter duration [7], lower 6MP dosage [8], inferior treatment adherence [9], and insufficient myelotoxicity [10, 11] have all been associated with increased risk of relapse. The importance of 6MP is further emphasized by the frequent acquisition of mutations driving thiopurine resistance in relapsed ALL [12–14]. Mutations in the post-replication mismatch repair system, which hamper cytotoxicity of 6MP, may also drive relapses [15, 16].

**Fig. 1:**
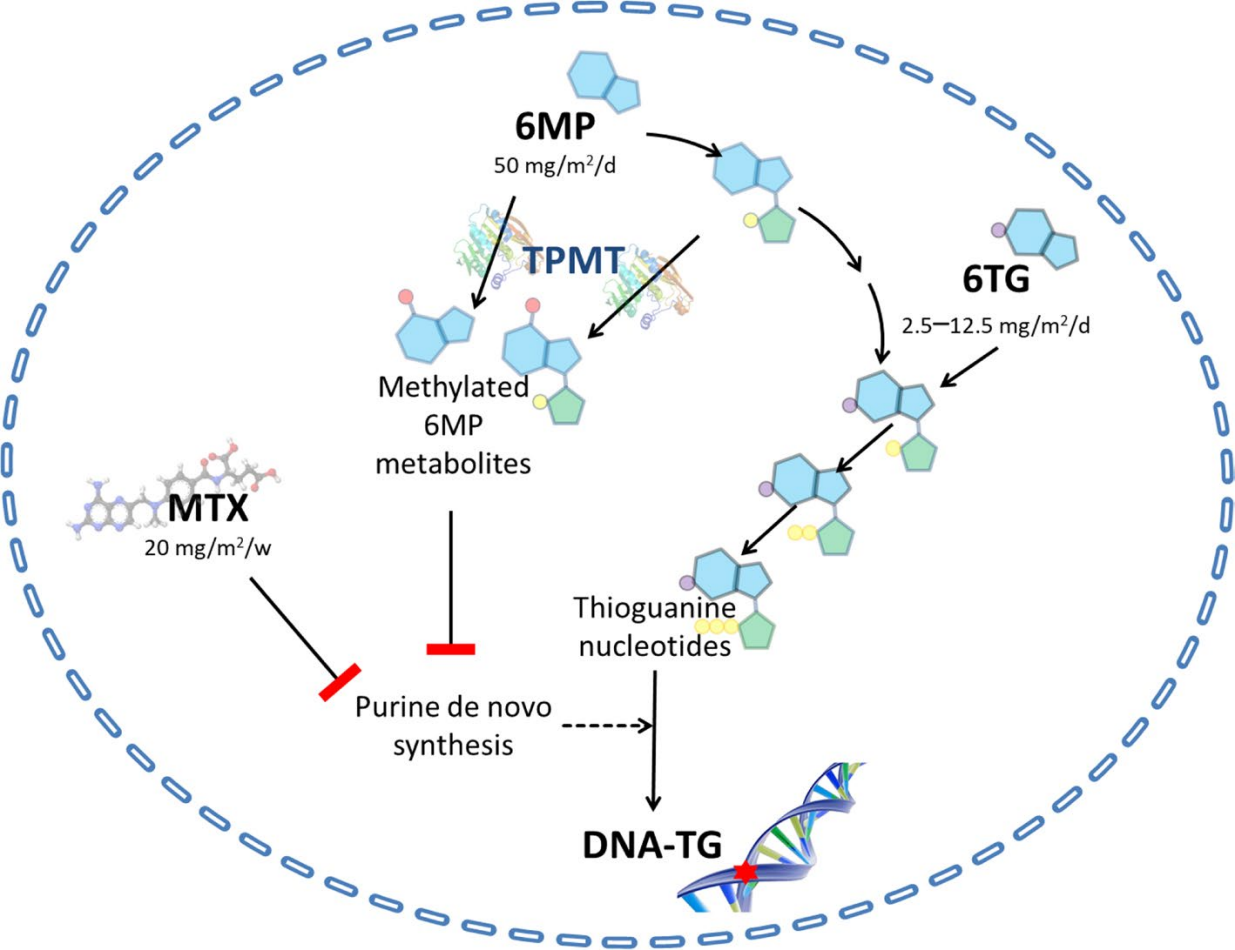
Intracellular metabolism of thiopurines and methotrexate. The tiopurines 6-mercaptopurine (6MP) and 6-thioguanine (6TG) are converted to thiguanine nucleotides (TGN) through sequential intracellular enzymatical steps. TGN are then incorporated into DNA (DNA-TG). The enzyme thiopurine methyl transferase (TPMT) creates methylated 6MP metabolites, which are associated with hepatotoxicity. Methotrexate (MTX) and some of the methylated 6MP metabolites inhibit purine de novo synthesis, thereby enhancing incorporation of TGN into DNA. Thus, DNA-TG is a downstream metabolite that integrates all upstream thiopurine and MTX metabolites

MTX and 6MP are prodrugs, and their cytotoxicity depends on intracellular metabolism. The primary cytotoxic endpoint of thiopurine therapy is incorporation of the 6MP metabolites thioguanine nucleotides (TGN) into DNA (DNA-TG) in competition with natural guanine [10]. DNA-TG occasionally becomes S-methylated and mismatches with thymidine, causing cell death after repetitive futile mismatch repair attempts [15]. A competing pathway for 6MP and several of its metabolites is S-methylation by thiopurine methyltransferase (TPMT), creating methylated 6MP metabolites (MeMP). MTX undergoes intracellular polyglutamation (MTXpg), enhancing intracellular retention as well as its effect on target folate reductase enzymes [17]. MTXpg and some MeMP are potent inhibitors of purine de novo synthesis, and can thus promote incorporation of TGN into DNA [10, 17–19] (Fig. 1). Monitoring as well as guiding maintenance therapy by erythrocyte levels of MTXpg, TGN or MeMP have been attempted, but have not been clearly associated with a reduced risk of relapse [8, 19, 20].

Hence, DNA-TG is a downstream metabolite integrating all upstream thiopurine and MTX effects (Fig. 1), and therefore, a method for monitoring DNA-TG in leukocytes has been developed [21]. The Nordic/Baltic NOPHO ALL2008 maintenance therapy study of 918 children with non-high risk ALL found a 10-fold interindividual difference in DNA-TG during maintenance therapy—thus, the individual mean DNA-TG ranged from 100 to more than 1000 fmol/μg DNA. Higher DNA-TG levels were significantly associated with a reduced risk of relapse in patients who were MRD positive at the end of induction (EOI), but this was not the case for EOI MRD-negative patients. Among the 526 EOI MRD-positive patients the relapse hazard was reduced by 28% (95% confidence interval (CI): 9–43%) for each 100 fmol/μg increase in DNA-TG (*p* = 0.0065) [20]. In NOPHO ALL2008 patients fulfilling the ALLTogether1 IR-high criteria, the relapse hazard was reduced by 39% (95% CI: 13–57%) for each 100 fmol/μg DNA increase in DNA-TG (*p* = 0.007) (Supplementary Table 1). As no significant associations were found between DNA-TG and 6MP dose, blood counts (neutrophils, lymphocytes, thrombocytes, hemoglobin), or aminotransferase levels during maintenance, these cannot replace DNA-TG measurements [20]. The association between DNA-TG and relapse hazard has been confirmed in a recent individual patient data (IPD) meta-analysis including the NOPHO ALL2008 pediatric cohort as well as five other international pediatric and adult ALL cohorts of patients aged 1–45 years [22]. Furthermore, DNA-TG was not associated with the hazard of developing a second malignant neoplasm (SMN) (hazard ratio = 0.88; 95% CI: 0.68–1.14; *p* = 0.34) [22].

It has recently been shown that significant DNA-TG increments can be obtained by adding a very low dose (2.5–12.5 mg/m^2^/day) of the thiopurine 6-thioguanine (6TG) to the MTX/6MP backbone of maintenance therapy [23]. The cytotoxicity of 6TG is also exerted through DNA-TG. However, 6TG is more extensively converted into TGN than 6MP, leading to 7-times higher erythrocyte TGN levels [24]; but as MeMP is lacking, a 6TG dose of 40 mg/m^2^ in combination with MTX does not cause profound myelosuppression [24], and it does not provide higher DNA-TG levels [25]. We therefore hypothesized that significant, but tolerable, DNA-TG increments can be obtained by adding a very low dose of 6TG to the conventional MTX/6MP maintenance therapy, which we hereby name the Thiopurine Enhanced ALL Maintenance (TEAM) strategy.

The feasibility and safety of the TEAM strategy has been tested in a pilot study of 34 patients [23]. The backbone of 6MP (starting dose of 50 mg/m^2^/day) and MTX (starting dose of 20 mg/m^2^/week) was supplemented with 6TG at an initial dose of 2.5 mg/m^2^/day, which was then increased in steps of 2.5 mg/m^2^/day at 2 weeks intervals up to a maximum dose of 12.5 mg/m^2^/day. The patients did not experience increased myelotoxicity or hepatotoxicity compared with historical controls from the NOPHO ALL2008 cohort, and it led to significantly higher DNA-TG levels when compared to historical data from the NOPHO ALL2008 maintenance sub-study (median DNA-TG on TEAM therapy: 764 fmol/μg vs 492 fmol/μg in NOPHO ALL2008 patients; *p* < 0.0001) [23]. Based on the Cox regression model from the NOPHO ALL2008 maintenance sub-study, this corresponds to a theoretical reduction in relapse hazard of 59% [20, 23].

Both 6MP and MTX are hepatotoxic, and can lead to elevated aminotransferase levels and hypoglycemic episodes, both of which have been associated with high levels of MeMP [26, 27]. Hepatic sinusoidal obstruction syndrome (SOS) (previously named veno-occlusive disease [VOD]) has been associated with the use of 6TG instead of 6MP in maintenance therapy of childhood ALL. In three randomized trials replacing 6MP with 6TG at doses of 40–60 mg/m^2^/day [28–30], 10–25% of patients receiving 6TG experienced SOS in two trials [29, 30] or moderate to severe thrombocytopenia in two trials [28, 30]. In a meta-analysis analyzing IPD from 4000 patients randomized in these three trials, the odds ratio for SOS between patients randomized to 6TG versus 6MP was 7.16 (95% CI: 5.66–9.06) [31]. The replacement of 6MP with 6TG did not result in an overall improvement of EFS [31]. Since replacing 6MP with 6TG also eliminates MeMP this will lead to less inhibition of purine de novo synthesis, which explains why DNA-TG levels do not increase even though cytosol TGN levels are higher with 6TG therapy [25]. This may also explain the lack of efficacy improvement in these trials.

A systematic review of hepatotoxicity during long-term use of 6TG in patients with ALL or inflammatory bowel disease concluded that both the occurrence and severity of 6TG-related hepatotoxicity appear to be highly dose-dependent and rarely occurs at doses below 12 mg/m^2^/day [32]. Using the TEAM strategy is therefore anticipated to be associated with a low risk of SOS. This conclusion is further supported by a retrospective study including 11 patients receiving 6TG who developed SOS and 121 who did not. In this study, SOS was not associatied with DNA-TG (hazard ratio = 0.91 [95% CI 0.76–1.09%; *p* = 0.30] per 100 fmol/μg increase in DNA-TG in a simple Cox regression) [25]. Furthermore, studies have shown angioprotective functions of 6MP possibly through the enhanced activity of the transcriptional factors Nur77 [33] and hypoxia-inducible factor-1α [34], and decreased expression of vascular adhesion molecules PECAM-1 and VLA-4 [35].

As MTX/thiopurine drug dosing will be titrated to the same degree of myelotoxicity, the TEAM strategy is anticipated to reduce relapse rate without significantly increased myelo- or hepatotoxicity. Still, study participation may potentially provide both harms and benefits. The TEAM protocol does not in itself prescribe more frequent hospital visits, which, however, may be necessary due to unexpected toxicities. However, the treating physician will be recommended to reduce the dose of 6TG if DNA-TG > 1500 fmol/μg DNA in two consecutive measurements within a 3-month period (but at least 2 weeks apart). The reduced 6MP dose (and thus reduced levels of MeMP) could lead to a lower frequency of hypoglycemia, nausea, and other hepatotoxicities associated with high levels of MeMP. For both study arms, MTX/6MP metabolite measurements may reveal poor adherence, which if subsequently improved may in itself increase cure rates.

In conclusion, the TEAM study hypothesizes that the risk of relapse can be significantly reduced without an excess of unacceptable toxicities by adding very low doses of oral 6TG to the 6MP/MTX backbone of maintenance therapy. The risk of death in first remission and SMN is expected to be the same in both treatment arms. Thus, the primary objective of the TEAM study is to significantly improve the 5-year disease-free survival (DFS) of IR-high ALL patients by a reduction in relapse rate. This should be achieved by adding 6TG to 6MP/MTX-based maintenance therapy. As 6TG drug dosing in general is not dependent on the measurement of DNA-TG or other MTX/thiopurine metabolites, the TEAM strategy can be widely implemented if shown to be of clinical benefit.

## Methods

### Study design

The TEAM study is a randomized, open label, multi-national phase 3 trial with ~ 5 years patient accrual and the end of the study is defined as the last patient’s last follow-up visit or 5 years from that patient’s end of induction therapy, whichever occurs first. Patients will be equally distributed between the control and the experimental TEAM arms.

### Study population

In the ALLTogether1 protocol two independent randomized treatment intensification studies are performed for IR-high patients (Randomization 3 [R3]: The R3-Inotuzumab (InO) and R3-TEAM sub-protocols). Thus, both the experimental arms will be independently compared to the control arm, but the experimental InO and TEAM arms will not be compared.

Eligibility for the two R3 studies is dependent on geography (United Kingdom (UK) vs non-UK), immunophenotype (BCP- vs T-ALL), inclusion criteria, and exclusion criteria. Table 1 summarizes the eligibility differentiating criteria with mandatory results before randomization. Patients may be randomized 2-way standard maintenance (SM) vs. InO + SM, 2-way SM vs. TEAM or 3-way SM vs. InO vs. TEAM, based on inclusion and exclusion criteria.

**Table 1:** Eligibility for the two Randomization 3 studies

Criteria	InO	TEAM
CD22-pos BCP-ALL	Yes	Yes
CD22-neg BCP-ALL	No	Yes
T-ALL	No	Yes
ALL prone syndromes^a^	Yes	No
TPMT/NUDT15 deficiency	Yes	No
United Kingdom	Yes	No
Known history of SOS/NRH	No	No
Cardiac function: SF < 30% / EF < 50%	No	No
Active systemic infection	No	No
Creatinine > 2 X UNL	No	No
ALT > 3 x UNL at inclusion	No	No
Bilirubin > 1.5 x UNL^b^	No	No
Pregnancy/Lactation/No CC	No	No
ABL-class fusion	No	No
BSA < 0.3 m^2^	No	No
Unable to take liquid medicine	Yes	No
Informed consent	Yes	Yes

*ALT* alanine aminotransferase, *BSA* body surface area, *CC* contraception, if indicated, *EF* ejection fraction, *InO *Inotuzumab, *SF* shortening fraction, *SOS* sinusoidal obstruction syndrome, *TEAM* Thiopurine Enhanced ALL Maintenance, *UNL* institutions’ upper normal limit. ^a^Exploration not mandatory. ^b^If total bilirubin > 1.5 x UNL, patients are still eligible if conjugated bilirubin ≤1.5 x UNL

The view of clinicians in the UK was that given the particular structures of clinical care for childhood ALL in the UK with a highly devolved process for the administration of oral maintenance chemotherapy with a very high number of shared care centres, that the safe and effective delivery of TEAM and collection of the requisite research data would be significantly challenged. This will be kept under review as the trial embeds into clinical practice. It is possible that a further amendment may be put forward for consideration for later inclusion of the UK in the TEAM study.

#### Population

Children and young adults (age 0–45 years) with newly diagnosed ALL treated according to the IR-high group of the ALLTogether1 protocol treated in Belgium, Denmark, Finland, France, Germany (COALL group centres), Estonia, Iceland, Ireland, Lithuania, the Netherlands, Norway, Portugal, and Sweden (for a list of study sites see Additional file 1).

#### Inclusion criteria

In order to be eligible to participate in the TEAM study, a subject must meet all of the following criteria:

- BCP-ALL (3-arm randomization) or T-ALL (2-arm randomization)
- Treatment according to the IR-high stratification group of the ALLTogether1 protocol
- Age at ALL diagnosis 0–45 years
- Written informed consent by the patient/legal guardian

At the time of randomization for TEAM all of the following criteria must also be fulfilled:

1. Serum creatinine < 2 x institutional upper normal limit (UNL).
2. Alanine aminotransferase (ALT) < 3 x institutional UNL.
3. Total bilirubin < 1.5 x institutional UNL. If total bilirubin is > 1.5 x institutional UNL, the patient is eligible, if conjugated bilirubin is < 1.5 x institutional UNL.

In patients for whom these parameters are above the limits but being normalized, randomization and start of maintenance therapy can be postponed after discussion with the international or the national principal investigator (PI). Later during maintenance therapy more abnormal parameters may indicate dose adjustments, but the patient will continue on the TEAM study.

#### Exclusion criteria

A potential study subject who meets any of the following criteria will be excluded from participation in TEAM (and the InO study):

- A history of prior or ongoing SOS.
- Cardiac function: shortening fraction < 30% by echocardiogram or an ejection fraction
  < 50% by multigated aquisition scan (MUGA).
- Active systemic infection (a systemic fungal, bacterial, viral or other infection with ongoing signs/symptoms related to the infection and without improvement despite appropriate antibiotics or other treatment).
- ABL-class fusions potentially sensitive to tyrosine kinase inhibitors.
- Body surface area (BSA) (using Mosteller BSA formula) < 0.3 m^2^ at randomization.
- Breast-feeding women.
- Sexually active female patients of childbearing potential with positive urine or serum pregnancy test prior to enrolment.
- Male and female patients of child-bearing potential who do not agree to use an effective method of contraception approved by the investigator during the study.

Additional exclusion criteria for TEAM only:

- Patients must have been screened for low activity TPMT alleles (and for low activity NUDT15 alleles, if of Asian ancestry). Patients with TPMT deficiency (defined as two low activity TPMT alleles or Ery-TPMT activity < 2.0 IU/ml) or NUDT15 deficiency (defined as two low activity NUDT15 alleles) are excluded. Patients who are compound heterozygous for both a low activity TPMT allele and a low activity NUDT15 allele are excluded.
- Known ALL prone syndromes (e.g. Li-Fraumeni syndrome, germline ETV6 mutation). Exploration for ALL prone syndromes is not mandatory.
- Patient not able to take liquid medicine.
- A history of prior or ongoing nodular regenerative hyperplasia (NRH) shown by biopsy.
- Intolerance to the substance or any of the expedients.

### Treatment of subjects

#### Investigational treatment

6TG is the primary investigational drug. As this drug is given in combination with and is titrated with 6MP and MTX, these drugs also become investigational drugs in the experimental TEAM arm (Table 2).

**Table 2:** Formulation, administration and toxicity of investigational drugs

Drug	Formulation	Administration	Toxicity
Methotrexate (MTX)	Tablets	Orally, once weekly.	Bone-marrow suppression leading to leucopenia, thrombocytopenia, and anemia. MTX is also hepatotoxic.
6-mercaptopurine (6MP)	Tablets and liquid formulation	Orally, daily. Doses are to be taken once a day at a regular schedule.	Bone-marrow suppression leading to leucopenia, thrombocytopenia, and anemia. 6MP is also hepatotoxic. The incidence of hepatotoxicity varies considerably and can occur with any dose, but more frequently when a dose of 75 mg/m^2^ body surface area per day is exceeded, and most frequent in *TPMT* wild type patients. In general, the incidence and severity of side effects are considered to be dose-related.
6-thioguanine (6TG)	5 mg/mL oral suspension^a^	Orally, daily. Doses are to be taken once a day at a regular schedule.	Bone-marrow suppression leading to leucopenia, thrombocytopenia, and anemia. 6TG is also hepatotoxic. At doses higher than those used in TEAM (40–60 mg/m^2^) 6TG has been related to an increased risk of sinusoidal obstruction syndrome and nodular regenerative hyperplasia.

^a^The oral suspension is provided by Nova Laboratories Ltd. to the TEAM study. The liquid preparation has a limited shelf life (see package). Only 40 mg 6-thioguanine tablets are presently marketed, making accurate dosing difficult to achieve, especially for treatments using doses of less than 20 mg. Although the currently approved tablet formulation can be halved or quartered to derive an intermediate dose, in most cases this still requires rounding doses up or down. Moreover, young children, many adolescents and some adults find taking tablets very difficult. Acceptability of the formulation is of paramount importance, especially in diseases where adherence can significantly impact on outcomes. Suspension administered using an oral syringe will allow the dose of 6-thioguanine to be tailored to patient requirements, both accurately and safely. Moreover, improved ease of administration for children is expected to enhance medication acceptability and adherence

The comparator is the standard maintenance therapy backbone with 6MP and MTX.

#### Reference safety information

For Reference Safety Information see the applicable SmPCs.

Since 6TG presently only has market authorisation as tablets, the SmPC for 6TG tablets is used as reference safety information for the oral suspension (Table 3).

**Table 3:** Summary of product caracteristics (SmPC) for 6-thioguanine

Genric name (abbreviation)		6-thioguanine (6TG)
ATC-code		ATC-code L0JBB03
Name and formulation		Oral suspension, 5 mg/ml
Adverse Reactions (frequency)	Very common (> 1/10)	Bone marrow failure, sinusoidal obstruction syndrome (SOS), hyperbilirubinemia, hepatomegaly, weight increase due to fluid retention and ascites, portal hypertension, splenomegaly, esophageal varices and thrombocytopenia, increased hepatic enzymes, increased blood alkaline phosphatase and gamma glutamyl transferase, jaundice, portal fibrosis, nodular regenerative hyperplasia, peliosis hepatis
	Common (> 1/100 to < 1/10)	Stomatitis, gastrointestinal disorder, SOS in short-term cyclical therapy, hyperuricemia, hyperuricosuria and urate nephopathy
	Uncommon (≥1/1000 to < 1/100)	
	Rare (≥1/10,000 to < 1/1000)	Necrotising colitis, hepatic necrosis
	Very rare (< 1/10,000)	
	Unknown	Photosensitivity

SmPC accessed at medicines.org.uk

#### Non-investigational products

All other drugs given as part of the ALLTogether1 IR-high treatment arm are given independently of the titration mentioned above and these are therefore not regarded as investigational treatments.

### Maintenance therapy

Maintenance therapy is initiated in treatment week 37 and continued in 4-week cycles until 2 years from complete remission (25 months/108 weeks from diagnosis) (Table 4). This duration is independent of the total cumulative administered number of days and drug doses. When initiating maintenance therapy, patients should fulfill the following criteria:

- Absolute neutrophil count (ANC) ≥0.75 × 10^9^/L (and not falling)
- Platelets ≥75 × 10^9^/L (and not falling)
- Serum creatinine ≤2 x the UNL
- Alanine aminotransferase (ALT) ≤ 3 UNL
- Bilirubin ≤1.5 x UNL
- No active systemic infection or evidence of hepatic SOS
- Cardiac shortening fraction ≥30% or ejection fraction be ≥50% by echocardiography/MUGA

**Table 4:** Mandatory examinations during maintenance therapy

Maintenance therapy week		0	4	8	12	24–36-48-etc
Timeline for standard and experimental arm	Screening d − 14 to 0	Start MT d1	2nd cycle MT d 29	3rd cycle MT d 57	4th cycle MT d 85	Every 12 weeks till end of maintenance
Informed consent^a^	X					
Medical History^b^	X	X	X	X		X
Physical Examination	X	X	X	X	X	X
Height, weight, BSA^c^	X	X	X	X		
Vital signs^d^	X	X	X	X		
Performance Status^e^	X	X	X	X		
Pregnancy test^f^	X		(X)	(X)	(X)	(X, monthly)
Echocardiogram	X^q^					X (end of MT)
Eligibility and registration^g^	X					
Concomitant Med	X	X	X	X	X	
Treatment						
LP with IT^h^				X		ALLTogether1
Maintenance therapy (6MP/MTX/6TG/VCR/DEXA)		Continuously				
Safety FU						
CBC and differential	X	X	X	X	X	X
Chemistry panel A^i^	X	X	X	X	X	
Chemistry panel B^j^						X
Amylase, Lipase	X			X		
Serum Ig levels^k^	X			X		X
Immunophenotyping of B-cells^l^		(X)			(X)	(X)
Adverse events	X	X	X	X	X	ALLTogether1
AESIs^m^	X	X	X	X	X	X
6MP/MTX metabolites^o^			X	X	X	X (monthly)
Efficacy FU						
Disease/survival status^p^		X			X	ALLTogether1

*BSA* body surface area, *CBC* complete blood count, *ECG* electrocardiography, *LP* lumbar puncture, *MRD* measurable residual disease, *IT* intrathecal therapy, *ALLTogether1* according to description in ALLTogether1 protocol

^a^ Informed consent must be obtained before any study specific investigations are performed Information can be handed out and consent can be obtained from treatment phase “IR-High Consolidation 2” onwards

^b^ Medical History including review of cancer diagnosis and previous cancer treatment (screening/pre-study visit only), current medications and any current medical conditions or abnormalities

^c^ Calculate BSA $$\left(\sqrt{\frac{\mathrm{Height}\left(\mathrm{cm}\right)\times \mathrm{Weight}\left(\mathrm{kg}\right)}{3600}}\right)$$

^d^ Vital signs include pulse, respirations, blood pressure and temperature

^e^ Performance status, see Additional file 2

^f^ Serum/Urine Pregnancy Test: For patients with child bearing potential, a serum or urine pregnancy test will be performed. If this is not indicated this should be documented in the patient file. Such monthly pregnancy testing must be done until 30 days after the end of maintenance therapy for women of child-bearing potential. Sexually active men must use an effective method of contraception until 90 days efter the end of maintenance therapy

^g^ Online eligibility and registration/ randomization

^h^ Intrathecal therapy will be given as SOC according to the ALLTogether1 protocol

^i^ Chemistry panel A: sodium, potassium, phosphate, creatinine, urea, albumin, total bilirubin (add direct bilirubin when total bilirubin is > UNL), AST, ALT, GGT and alkaline phosphatase

^j^ Chemistry panel B: AST, ALT, total bilirubin (add direct bilirubin when total bilirubin is > UNL), creatinine

^k^ Serum IgG (at screening IgG, IgA IgM)

^l^ Immunophenotyping of B-cells is optional. Standard B cell markers include CD19, CD27, CD38, CD10 as well as stainings for IgM, −G,-A and –D

^m^ AESIs: incidence of > grade 3 infections, IgG levels/administration of IVIG, SOS all grades, ALT and bilirubin > grade 3 are collected from start of maintenance until end of maintenance

^o^ 6TG/6MP/MTX metabolites (Send to Rigshospitalet, Copenhagen Denmark). Samples must be sent at least every 3 months, and are recommended to be sent monthly

^p^ Disease and survival status: Continuous CR, Relapse (including molecular relapse), Second malignant neoplasm, death/cause of death

^q^ Screening echocardiogram can performed during consolidation 3, provided that written informed consent for randomisation R3 has been obtained. When an echocardiogram has been performed after the last dose of doxorubicin in delayed intensification, but before the screening period there is no need to repeat an echocardiogram for screening

### Control arm maintenance therapy

Initial 6MP dose of 75 mg/m^2^/day orally. In patients with homozygous NUDT15 or TPMT-deficiency, the starting 6MP dose should be reduced to 5 mg/m^2^. Initial MTX dose of 20 mg/m^2^/week orally. During the weeks when intrathecal therapy is given, the oral MTX dose is omitted or adjusted to an appropriate total dose depending on the size of the patient. Maintenance therapy is continued until the end of therapy (25 months/108 weeks from diagnosis).

Dexamethasone (Dexa)/Vincristine (VCR) pulses every 4 weeks throughout maintenance, given regardless of blood counts. One pulse consists of: 1) 6.0 mg/m^2^/day Dexa orally for 5 days, divided into three daily doses (2.0 mg/m^2^/dose). No tapering. May influence neutrophil counts, which should not lead to maintenance therapy dose adjustments; 2) 1.5 mg/m^2^ VCR (maximum single dose 2.0 mg) intravenously on day 1 of the pulse. It is allowed to move the VCR-dose 1 day forward to avoid giving it the same day as the intrathecal therapy.

Intrathecal therapy (IT) with single MTX or Cytarabine/Methotrexate/Steroid (ITT) according to CNS-status at diagnosis and early response, dosing by age according to the ALLTogether1 protocol, given every 12 weeks throughout maintenance up to a total of six doses.

#### 6MP/MTX dose adjustments

Maintenance doses must be adjusted to keep the ANC level between 0.75–1.5 × 10^9^/L and platelets above 75 × 10^9^/L.

Due to interindividual variations in pharmacokinetics and drug tolerance the dosage needed during maintenance to obtain the target myelosuppression may vary significantly among patients.

Dose adjustments can follow the national guidelines or the optional ALLTogether1 guidelines on maintenance therapy.

### Team experimental arm maintenance therapy

Initial 6MP dose of 50 mg/m^2^/day orally. NB. This dose is 2/3rd of the starting dose in the control arm to avoid excessive inhibition of purine de novo synthesis mediated by MeMP. Initial MTX dose of 20 mg/m^2^/week orally. During the weeks when intrathecal therapy is given, the oral MTX dose is omitted or adjusted to an appropriate total dose depending on the size of the patient. Maintenance therapy is continued until the end of therapy (25 months/108 weeks from diagnosis).

Initial 6TG dose is 2.5 mg/m^2^/day with dose increments of 2.5 mg/m^2^/day at 2-week intervals until the maximum tolerated dose of 6TG is reached. The maximum dose of 6TG is set to 12.5 mg/m^2^/day for all patients throughout maintenance therapy.

Dexa/VCR pulses as described for the control arm. During maintenance therapy, patients will be seen for clinical examination and blood sampling at least every 4 weeks. During the initial 6TG dose increment period, the interval will be every 2 weeks.

#### 6TG/6MP/MTX dose adjustments

Maintenance doses must be adjusted to keep the ANC level between 0.75–1.5 × 10^9^/L and platelets > 75 × 10^9^/L.

Throughout maintenance therapy a 6TG dose of 12.5 mg/m^2^ should be attempted, unless the the patient has been unable to tolerate this without toxicities. This is also the case for patients with ANC within the target range of 0.75–1.5 × 10^9^/L. If an ANC of 0.75–1.5 × 10^9^/L is not reached with the maximum dose of 6TG of 12.5 mg/m^2^/day, dose increments of 6MP and MTX (at unchanged 6TG dosage) are recommended as outlined below.

High ANC: If ANC subsequently (i.e., at a 6TG dose of 12.5 mg/m^2^) is > 1.5 × 10^9^/L and platelets > 100 × 10^9^/L, the dose of 6MP should be escalated in steps of 25% up to 150 mg/m^2^/day. If the ANC is still ≥1.5 × 10^9^/L at a 6MP dose of 150 mg/m^2^/day, increase oral MTX by 25% (from 20 mg/m^2^/week) up to a dose of 40 mg/m^2^/week. Consider non-adherence in case of tolerated doses of 6MP above 125 mg/m^2^/day and of MTX above 30 mg/m^2^/week, when dose of 6TG is 12.5 mg/m^2^/day. In principle, there are no maximum doses for 6MP and MTX, but persistent and tolerated doses of 6MP above 150 mg/m^2^/day or MTX above 40 mg/m^2^/week should be discussed with the national principal investigators of the ALLTogether1 and the TEAM study. The dose of 6TG should never be higher than 12.5 mg/m^2^/day.

6TG dose < 12.5 mg/m^2^/day: If the patient after the first 2 months of maintenance therapy is receiving 6TG at a dose < 12.5 mg/m^2^/day, and the patient has ANC > 0.75 × 10^9^/L and platelet count > 75 × 10^9^/L, upward dose adjustment of 6TG should be attempted with dose increments of 2.5 mg/m^2^/day at *4 weeks intervals* to a maximum of 12.5 mg/m^2^/day.

Neutro- and/or thrombocytopenia, moderate: If the neutrophil count falls to 0.5–0.75 × 10^9^/L and/or the platelet counts falls to 50–75 × 10^9^/L within 4 weeks from 6TG increment, then reduce 6TG by 2.5 mg/m^2^/day, otherwise halve the dose of 6MP, 6TG, and oral MTX.

Neutro- and/or thrombocytopenia, severe: If the ANC falls to < 0.5 × 10^9^/L and/or the platelet count < 50 × 10^9^/L: STOP 6MP, 6TG, and oral MTX. RESTART at the tolerated dose (= before treatment interruption), when ANC > 0.75 × 10^9^/L and platelet count > 75 × 10^9^/L, but reduce 6TG by 2.5 mg/m^2^/day if the neutro- and/or thrombocytopenia occurred within 4 weeks from a 6TG dose increment. If counts fluctuate wildly after 6MP and MTX are reintroduced and cross the limits for treatment interruption, then start at 50% of the previously tolerated doses and titrate upwards (first 6TG, then 6MP, then MTX) to avoid frequent interruptions of maintenance therapy.

In patients that develop thrombocytopenia < 75 × 10^9^/L not associated with neutropenia, SOS, NRH, or hypersplenism by other causes should be considered.

### Organ toxicities during maintenance therapy

Applicable to both TEAM and control arms.

ALT: During maintenance therapy ALT should be measured according to Table 4. A rise in ALT levels, even at 20–40 x UNL, does not in itself warrant dose adjustment. Low ALT is seen in 5–10% of patients, who have low TPMT activity. These patients generally respond to 6MP dose increments with neutropenia.

Bilirubin: Measurements of bilirubin should be done according to Table 4. If direct bilirubin is > 3 x UNL, 6TG/6MP/MTX dosing should be reduced or withheld. Other causes of hyperbilirubinemia should be considered, e.g., viral hepatitis, SOS, or Gilbert syndrome.

Coagulation factors: Measurements of coagulation factor II-VII-X should be done every 3 months. If coagulation factors II-VII-X fall below 0.5, oral 6TG/6MP/MTX should be reduced or withheld. Consider other causes than 6TG/6MP/MTX therapy (e.g., viral hepatitis, K-vitamin deficiency). If coagulation factors are the only sign of hepatotoxicity, consider K-vitamin supplementation.

Hypoglycemia: Hypoglycemia (not least after fasting, incl. in the morning) is frequently seen during maintenance therapy due to impaired glycogenolysis and/or gluconeogenesis. Some patients complain of fatigue, nausea, or gastrointestinal symptoms. They generally have elevated ALT levels, but otherwise normal liver function tests. Neither these symptoms nor hypoglycemia in itself indicate dose adjustments. Some patients respond well to diabetic food (slowly absorbed carbohydrates) and/or rapidly absorbed carbohydrates (e.g., apple juice), when having symptoms. Severe, refractory cases should be discussed with the national principal investigators of ALLTogether1. Neither these symptoms nor hypoglycemia in itself indicate 6TG/6MP/MTX dose adjustments.

Sinusoidal obstruction syndrome (SOS): In case of persistent discordant thrombocytopenia < 75 × 10^9^/L (i.e., not accompanied by neutropenia < 0.75 × 10^9^/L) or any of the other clinical indications of SOS, the patient should be evaluated for SOS, incl. abdominal ultrasound, to determine the presence of ascites, reduced liver vessel flow, hepatic steatosis or fibrosis. The true incidence of SOS during 6MP/MTX therapy combined with very low dose 6TG is unknown. SOS is defined according to the international Ponte di Legno definition, fulfilling at least 3 of 5 criteria: (1) Hyperbilirubinemia, (2) unexplained thrombocytopenia (i.e., without concurrent neutropenia), (3) weight gain > 5.0%, (4) liver pain, and (5) ascites. Sonographic demonstration of compromised hepatic blood flow can support the diagnosis, but is frequently normal. In case of SOS in a patient in the experimental TEAM arm, that patient is taken off 6TG and treated according to the 6MP/MTX control arm. Any case of SOS should be registered in the ALLTogether1 online database within 24 h.

Nodular regenerative hepatotoxicity (NRH): This is a non-cirrhotic portal hypertension, which can be induced by thiopurine therapy. There are typically symptoms of portal hypertension (weakness, ascites, splenomegaly, esophageal varices) in a patient with little evidence of chronic liver disease. The diagnosis is confirmed by liver biopsy, showing absence of significant fibrosis and presence of nodularity usually best defined by reticulin staining. On superficial review, the liver biopsy may appear normal. NRH is usually accompanied by unexplained thrombocytopenia. Serum enzymes are generally normal or minimally elevated, although acute elevations in serum enzymes accompanied by SOS may precede the development of NRH. Jaundice is rare. Ultimately, NRH may lead to liver failure and need for liver transplantation. Among ALL patients, NRH has mostly been described with 6TG therapy, but it is very rare with 6MP therapy or low dose 6TG therapy (< 12.5 mg/m^2^). In case of NRH in a patient in the experimental TEAM arm, that patient is taken off 6TG and then treated according to the 6MP/MTX control arm. Any case of NRH should be registered in the ALLTogether1 online database within 24 h.

Skin rashes: Skin rashes as an adverse drug reaction can be seen during maintenance therapy, but is far more common 2–6 weeks after cessation of maintenance therapy. It usually disappears without any intervention. MTX/6MP/6TG therapy should be continued. The rash might persist, but does not pose a problem.

### Blood sampling for thiopurine/MTX metabolite analyses

Blood samples for DNA-TG measurement are recommended to be sent monthly, but intervals up to 3 months are acceptable and obligatory in all three arms of the R3 randomization.

Blood samples: An EDTA anticoagulated blood sample of 3 ml is required. If micro/capillary samples are taken, a minimum of 1 ml is needed for DNA-TG analysis.

Shipment: Blood samples are sent by standard mail to University Hospital Rigshospitalet, Bonkolab 5704, Denmark. Alternatively, samples can be frozen locally and sent once a month. Analyses are done free of charge.

Low Ery-TGN, Ery-MeMP, Ery-MTX: If Ery-TGN and/or Ery-MeMP and/or Ery-MTX are low, these results will be reported back to the treating physician, who can judge whether this is due to known recent treatment interruption (e.g., organ toxicity or infections) or if it should raise suspicion of poor treatment adherence, which can then be discussed with the patient/family.

DNA-TG > 1500 fmol/μg is rarely seen during 6MP/MTX maintenance therapy. If DNA-TG for a patient in the TEAM experimental arm is > 1500 fmol/μg DNA at two consecutive measurements during a 3-month period (but at least 2 weeks apart), the 6TG dose should be reduced by 2.5 mg/m^2^/day.

Sample storage: Blood samples will be analyzed shortly after arrival at Rigshospitalet, Copenhagen (> 95% within 1 month after arrival) and stored until analysis. The storage will be submitted for approval by the Danish Data Protection Agency, since this is required if research samples are stored for more than 8 days. The samples will be discarded or returned to their local (or national) biobanking facility when all analyses have been performed.

#### Preparation and labelling of investigational medicinal product

Preparation and labelling of the investigational medicinal products will be done according to the relevant national GMP guidelines. See annex 13 of the guideline Good Manufacturing Practice (GMP) (2003/94/EG, via http://ec.europa.eu/health/files/eudralex/vol-4/2009_06_annex13.pdf).

The study drug ‘Tioguanine 5 mg/mL oral suspension’, developed by Nova Laboratories Ltd., will be provided by the TEAM study center in Copenhagen. The storage and distribution of the study drug will be managed by KLIFO A/S, Glostrup, Denmark. KLIFO will re-label bottles and cartons with booklet labels in local languages. Both labels will carry a unique kit number, to make it possible to identify all bottles.

#### IMPs that are also drugs of standard care

The Investigational Medicinal Products (IMPs) 6MP and MTX are identified only by active substance and will be sourced locally from the market of each participating country. These IMPs will not be provided by the sponsor. In accordance with the ALLTogether1 protocol, exemptions from study-specific labelling will be sought for these standard-of-care drugs.

#### Drug accountability

KLIFO is responsible for the overall drug accountability. Nova Laboratories Ltd. will be responsible for release of drug substance and drug product. KLIFO will perform final batch certification, and provide shipping information on a kit level. Bulk and packed product will be stored at KLIFO at 15–25 °C according to specifications at Smedeland 36, DK-2600 Glostrup, Denmark. KLIFO will deliver the study drug to the approximately 100 trial sites in Europe in temperature controlled shipments. These local trial sites will be responsible for drug accountability including re-distribution of 6TG to their shared care centers. Thus, each site must maintain adequate records documenting the inventory and disposition of all 6TG received, used and unused during the course of this clinical trial. The 6TG supplied for this trial is for investigational use only and to be used only within the context of treating patients formally enrolled onto this clinical trial by authorized personnel experienced in handling cytotoxic therapies.

Responsibility for 6TG accountability at the trial sites rests with the PI of the institution. The PI and/or a pharmacists or other appropriate individual, who is designated by the PI, should maintain records of 6TG delivery to the trial site, the inventory at the site, the use by each subject, and the return and destruction of unused product. These records should include dates, quantities, batch numbers, expiration dates, and the unique code numbers assigned to the 6TG and trial subjects. Investigators should maintain records that document that the subjects were provided the doses specified by the protocol and reconcile all 6TG received.

The PI should ensure that 6TG is stored at the treating centre as specified by sponsor and in accordance with applicable regulatory requirements. The PI should ensure that the investigational products are used only in accordance with the approved protocol.

The PI, or a person designated by the PI, should explain the correct use of the investigational products to each subject/family and should check, at appropriate intervals, that each subject is following the instructions properly.

To ensure adequate records, all study drug must be accounted for on drug accountability forms. Used or partially used bottles should be destroyed at the site and accurate records must be kept and made available to trial personnel on request and during monitoring visits. Unless otherwise authorized by trial personnel, at the end of the clinical study all 6TG supplies unallocated or unused must be destroyed according to the center’s own practice, or alternatively returned to KLIFO.

KLIFO will archive all documentation related to import and release of the study drug for 10 years, hereafter sponsor will be contacted to decide on further archiving of the documentation. Nova Laboratories Ltd. will retain batch manufacturing documentation in accordance with EU GMP requirements.

### Endpoints

#### Main study endpoint

5-year probability of DFS by intention-to-treat analysis.

#### Secondary study endpoints

- OS by intention-to-treat (including association with DNA-TG).
- Association of DFS with DNA-TG.
- Risk of relapse (including association with DNA-TG). Here death in first complete remission (DCR1) and SMN will be analyzed as competing events.
- Risk of SMN (including association with DNA-TG). Here DCR1 and relapse will be analyzed as competing events.
- Cumulative incidence of SOS and NRH.
- Cumulative incidence of osteonecrosis.

#### Other study parameters

- 6MP and MTX metabolite pharmacokinetics (i.e., Ery-TGN/MeMP/MTXpg).
- Abnormal liver function parameters (including hypoglycemia).

#### Randomization and treatment allocation

Screening is only allowed after written informed consent and can start 1–2 weeks after start of the last high-dose MTX in consolidation III. Screening after 2 weeks is preferred to prevent screening failures because of increased ALT, bilirubin or creatinine after high-dose MTX. 6MP will be stopped 2 weeks after start of HD-MTX. After screening, patients can be randomized as soon as they meet the inclusion criteria and do not meet any exclusion criteria.

Randomization will be using minimization stratified by: country, sex, age, WBC, immunophenotype, MRD and genetic risk (high-risk genetic changes, TEL/AML1, high hyperdiploidy, B-other). During the study (i.e., until all randomized patients are off therapy), the randomization code will be broken to the study statistician to provide the annual progress report to the Data and Safety Monitoring Board (DSMB).

#### Study procedures

- Blood samples will be taken as described.
- Events (relapse, DCR1, SMN) will be registered prospectively as for the total ALLTogether1 protocol.
- Toxicity capture: Toxicities will be registered prospectively as for the total ALLTogether1 protocol.

#### Withdrawal of individual subjects

Subjects can leave the study at any time for any reason, if they wish to do so. They will then be shifted to standard maintenance therapy for their remaining treatment. Except for the shift to standard maintenance therapy for those allocated to the experimental TEAM arm, their decision will not otherwise have any consequences for their further therapy. They will continue to be included in the intention-to-treat outcome analysis. In the per protocol analysis, they will be included until the day they withdrew from study participation. Patients in the experimental TEAM arm, who are shifted from the TEAM therapy to standard maintenance therapy due to unacceptable toxicity (incl. SOS), will remain in the intention-to-treat outcome analysis.

#### Replacement of individual subjects after withdrawal

Patients will not be replaced after withdrawal after randomization.

#### Premature termination of the study

The study can be prematurely terminated if the DSMB recommends this based on the annual progress reports on frequency of toxicities or relapse rate in the TEAM experimental arm. In such cases, all patients on the TEAM experimental arm will be shifted to control arm maintenance therapy.

### Safety reporting

#### Temporary halt for reasons of subject safety

The sponsor will suspend the study if there is sufficient ground that continuation of the study will jeopardize subject health or safety. The sponsor will notify the accredited regulatory authorities (ethical committee, medicine agency) without undue delay of a temporary halt including the reason for such an action. The study will be suspended pending a further positive decision by the accredited regulatory authorities. The investigator will make sure that all subjects are informed.

An interim report will be prepared and submitted to the DSMB once a year. The potential risk of SOS will be balanced against DFS. The former develops during therapy, whereas most relapses and SMNs emerges after the end of therapy. Since the proportional reduction in relapse rate is anticipated to be ~ 50%, the acceptable risk of SOS is set to 3%. If a patient develops SOS on the standard MTX/6MP arm, the local physician will decide whether the standard MTX/6MP therapy regimen can continue.

#### Adverse events (AEs) and adverse events of special interest (AESIs)

AEs are defined as any undesirable experience occurring to a subject during the study, whether or not considered related to the TEAM experimental arm.

A number of toxicities are so well-known and frequent during conventional 6MP/MTX maintenance therapy that they are to be expected and will not be considered AEs, and will not be reported routinely to the national regulatory authorities or the DSMC.

These include:

- Myelosuppression. Since this is the target of maintenance therapy, leucopenia and thrombocytopenia will not be regarded as an AE. This includes febrile neutropenia leading to hospitalization.
- A rise in ALT with normal liver function tests (i.e., bilirubin and INR (or factor II-VII-X)).
- A rise in bilirubin to less than 5 x UNL, except when SOS is diagnosed (if patients experience ALT at least 3 x UNL with bilirubin 2 x UNL within the first 3 months of maintenance therapy this will be reported).
- A decrease in coagulation factors II-VII-X.
- A rise in amylase of less than 5 x UNL.

Most of the toxicity-reporting will be carried out by the reporting of 23 predefined AESIs via the ALLTogether1 eCRF-system at specified time points defined by risk group. Most of these toxicities will not be reported as SAEs.

AESIs specifically related to TEAM are:

- Symptomatic osteonecrosis
- Liver failure with encephalopathy
- SOS/NRH
- Hypoglycemia leading to hospital admission

In addition these toxicities will be collected at the same time-points as the AESIs:

- Incidence of ≥ grade 3 infections
- IgG levels and administration of IVIG
- ALT and bilirubin elevations ≥ grade 3 (> 3 x UNL for bilirubin and > 5 x UNL for ALT)

In accordance with the ALLTogether1 protocol, expected AEs will not be reported for most of the study-period. However, for the first 12 weeks, AE-reporting will be more extensive: All AEs ≥ grade 3 reported spontaneously by the subject or observed by the investigator or staff will be recorded from the start of maintenance for the first 3 maintenance blocks (= 12 weeks) except for non-reportable AEs:

- Alopecia, fatigue, anorexia
- Laboratory abnormalities > grade 3 not considered to be clinically significant and/or responding to standard medical management

Abnormal laboratory findings without clinical significance (based on the investigator’s judgement) are not to be recorded as AEs. Clinical significance is defined as laboratory value changes that require clinical intervention or changes or adjustment in current therapy. Where applicable the clinical sequelae (not the laboratory abnormality) are to be recorded as the AE.

#### Serious adverse events (SAEs)

A serious adverse event is any untoward medical occurrence or effect that

- results in death
- is life threatening (at the time of the event)
- requires hospitalization or prolongation of existing inpatients’ hospitalization
- results in persistent or significant disability or incapacity
- is a congenital anomaly or birth defect, or
- any other important medical event that did not result in any of the outcomes listed above due to medical or surgical intervention, but could otherwise have been based upon appropriate judgement by the investigator.

An elective hospital admission will not be considered as a SAE.

The investigator will report all SAEs to the sponsor without undue delay (within 24 h) after obtaining knowledge of the events. However, this applies only to the first 12 weeks of the maintenance phase for both the experimental- and the control arm.

In addition to the SAE-criteria listed above, suspected serious drug-induced liver injury (Hy’s law cases – described in Additional file 3), should also be reported within 24 h for the first 12 weeks.

From the 13th week of the maintenance phase until the end of therapy only SOS/VOD and NRH should be reported within 24 h of obtaining knowledge of the event.

#### Suspected unexpected serious adverse reactions (SUSARs)

Unexpected adverse reactions are SUSARs, if the following three conditions are met:

1. The event must be serious.
2. There must be a certain degree of probability that the event is a harmful and an undesirable reaction to the medicinal product under investigation, regardless of the administered dose.
3. The adverse reaction must be unexpected, that is to say the nature and severity of the adverse reaction are not in agreement with the product information as recorded in:

- Summary of Product Characteristics (SmPC) for an authorized medicinal product.
- Investigator’s Brochure for an unauthorized medicinal product.

The sponsor will promtly forward via e-mail the SUSAR to the National Principle Investigator and/or Clinical Trial Unit (CTU) where the SUSAR occurred. The applicable country will report the SUSAR to the National Compent Authorities, web portal Eudravigilance and institutional review borad/ethics committee (IRB/EC) if applicable. In addition, the sponsor will promptly notify all NPIs/CTUs about a SUSAR. The sponsor has to report the SUSAR promptly to the NPIs/CTUs to enable the country to comply with the regulatory requirements and timelines for SUSAR reporting. The timeline to report the SUSAR is 7 days of first knowledge of the SUSAR that result in death or are life threatening followed by a period of maximum of 8 days to complete the initial preliminary report. All other SUSARS will be reported within a period of maximum 15 days after the sponsor has first knowledge of the SUSAR.

SUSARs are recorded in an overview list (line-listing) that will be submitted once every year to the accredited regulatory authorities. This line-listing provides an overview of all SUSARs from the study medicine, accompanied by a brief report highlighting the main points of concern.

#### Developmental safety update report (DSUR)

In accordance with the ALLTogether1 protocol, the sponsor will submit a safety report to the NPI/CTU in the participating countries once a year throughout the clinical study, to enable them to report the DSUR to the accredited IRB/EC, and competent authorities of the participating countries.

This safety report consists of:

- A list of all suspected (unexpected or expected) serious adverse reactions, along with an aggregated summary table of all reported serious adverse reactions, ordered by organ system.
- A compilation of AESIs for the whole patient-population and the pooled patient-populations for the risk-groups and for the randomized studies.
- A report concerning the safety of the subjects, consisting of a complete safety analysis and an evaluation of the balance between the efficacy and the harmfulness of the interventions under investigation.

The data in the annual progress and safety report is aggregated without specification of randomized arms.

#### Follow-up of adverse events

All AEs during the TEAM study will be followed annually until they have abated, or until a stable situation has been reached. Depending on the event, follow up may require additional tests or medical procedures as indicated, and/or referral to a general physician or a medical specialist.

#### Data safety monitoring board (DSMB)

The DSMB for the TEAM study will be the same as for the ALLTogether1 protocol. The DSMB periodically (at least annually) reviews safety and study data and makes recommendations based on their review along with assessing the performance of overall study operations and any other relevant issues, as necessary. The basis for the review is a report provided by the study statistician without the knowledge or influence by the sponsor, PI or steering committee. The DSMB will receive annual progress reports on patient recruitment, rate of events (relapse, DCR1 and SMN), occurrences of SOS, NRH, AESI, SAEs, SUSARs and levels of DNA-TG in the randomization arms.

The DSMB can recommend extension of the study to ensure adequate enrollment.

The advice of the DSMB will be sent to the sponsor of the study to be shared with the steering committee. Should the sponsor decide not to fully implement the advice of the DSMB, the sponsor will send the advice to the reviewing accredited competent authorities, including a note to substantiate why (a part of) the advice of the DSMB will not be followed.

### Statistical analysis

#### Sample size calculation

Historic data was pooled from four of the study-groups (UKALL, DCOG, COALL and NOPHO) of patients with positive EOI MRD-values consistent with the IR-group. This group was, by way of threshold analysis, sub-divided into an IR-Low and an IR-High risk group. The IR-high risk group was considered to have an unacceptably low 5-year DFS due to relapses (0.844), after consideration of events before the protocol time for the TEAM randomization (9 months).

#### Outcome assumptions

The TEAM- and InO sub-protocols (that run in parallel) are regarded as fully independent experiments. For each of the two studies the outcome of the experimental intervention will be compared to the control arm, but comparisons between the two experimental arms will not be made. The patient numbers and power calculations are based on:

- United Kingdom will not provide patients to the TEAM study.
- French centers will join the ALLTogether1 protocol from October 2021.
- There will be an 80% participation rate of all eligible patients for the TEAM study.
- Patient accrual will be uniform over the 5 years accrual period.
- 24% of randomized patients have T-ALL.
- 50% are randomized to the experimental TEAM for both T- and BCP-ALL.
- The 5-years DFS for control BCP-ALL patients is 84% and for T-cell ALL it is 81%.

The primary endpoint is DFS, where we aim to show an improvement in the 5-year rate from 84 to 91% for BCP-patients (HR 0.54) and 81 to 87% (HR 0.61) for T-cell ALL compared to standard maintenance. A minimum of 778 randomized patients would be appropriate to assess this improvement with 80% power and a 2-sided 5% alpha. With 878 patients recruited over 5 years (assuming 39% are IR-high and 80% accept randomization), an additional 5 years follow-up, a 2-sided 5% alpha we will have 84% power to detect this difference.

Recruitment into R3 – TEAM will be discontinued after 778 patients have been randomized and found to be evaluable. Since power has been calculated for a 5-year recruitment time and a minimum of 2 years follow-up time for the last recruited patient, the follow-up time will be extended if the recruitment time is significantly shorter than 5 years.

#### Analysis of endpoints

DFS will be calculated from date of randomization until date of death (from any cause), relapse or diagnosis of a SMN (whichever occurs first). Patients without an event will be censored at the date last seen. Analyses will be by intention-to-treat, with patients only excluded if they are later found to have been ineligible at randomization. The Kaplan-Meier method will be used for estimation of the 5-year DFS. The hazard ratio comparing DFS between the two treatment arms will be calculated from a Cox proportional hazards regression model and equality of hazards assessed with a Wald test, if the proportional hazards assumption is not violated. The Cox model will include the stratification variables used in the minimization: country, sex, age, WBC at diagnosis, MRD and genetic risk (high-risk genetic changes, TEL/AML1, high hyperdiploidy, B-other). The assumption of proportional hazards will be assessed using Schoenfeld residuals, and if violated the primary analysis will report the varying hazard ratio over the follow-up time along with their 95% confidence intervals.

The secondary endpoints will be measured from the date of randomization until date of event of interest and estimated using the Aalen-Johansen estimator. Differences for the randomized arms will be compared using Gray’s test. Patients who have not experienced the event of interest at the time of analysis will be censored at the date last seen, and the alternative secondary endpoints will be considered competing events. OS will be analysed using Cox regression and the Kaplan-Meier method will be used for estimation of 5-year OS.

### Ethical considerations

#### Regulation statement

The TEAM study will be conducted accord**i**ng to the principles of the Declaration of Helsinki (2008 version; www.wma.net) and in accordance with the Medical Research Involving Human Subjects Act, as well as the ICH Harmonised Tripartite Guidelines for Good Clinical Practice with applicable local and European regulations.

Patients will only participate when oral and written consent have been provided by the patient or their legal guardians by proxy, and will give their informed consent without their decision affecting the medical care they receive. All study subjects will be informed of their right to immediately leave the study, should they wish to do so, with no consequence to the medical care they receive. The protocol and proposed informed consent forms (Additional files 4 and 5) will be reviewed and approved by an IRB/EC in each participating country before the study commence. Approved by the Swedish Ethical Review Authority (No. 2020–04484).

Children are per legal definition incompetent with regards to giving informed consent. However, it is essential that this project is carried out including both children and adult patients with ALL as there are substantial differences in leukemia biology, drug metabolism, and serologic parameters between adults and children. This means that the toxicity profile and therapy effect can be very different between children and adults and necessitate the inclusion of children in this trial to achieve valid results for assessing the trial therapy in children with ALL. It is the legal guardians of the child who are to give consent of study participation; however, the opinion of the child will be respected, as it is crucial that the participants are sufficiently motivated. Study participation does not by itself demand extra visits to the hospital or extra blood samples unless an adverse event occur.

The rights, safety and well-being of the study subjects are the most important considerations, and will always prevail over interests of science and society. To ensure the safety of the study-subjects, interim results will be regularly (once yearly) reported to the DSMB, who will scrutinize the results and make an assessment if the possible gains by the study are still worth the calculated risk of the experimental therapy. This should be seen as both a scientific and an ethical safeguard for the participating patients.

This sub-protocol is initiated by coordinating investigator professor Kjeld Schmiegelow, Copenhagen, Denmark, and is supported by the ALLTogether1 steering committee. The study group has no conflicting political or financial affiliations.

Enrolled patients will not receive any economic compensation for participation in this study. Compensation for therapy-related injuries or adverse events will be covered according to the local or national regulations and not by the sponsor.

#### Recruitment and consent

Patients/guardians will be informed verbally and in writing about the TEAM study, randomization, and potential benefits and risks by the physician responsible for the patient’s ALL treatment or by a research nurse.

The local PIs will identify eligible trial subjects at the participating centers. Participants and/or their parents must be provided with the ″Written Information for Trial Subjects″ and oral information of the clinical trial at least 2 days before formal registration to ensure participants, and their parents or legal guardians, time for careful consideration of study participation. The PI (a physician with pediatric oncology experience if the participant is a minor) provides the oral information in an undisturbed setting, e.g., in a room exclusively with participation of the patient, the parents/legal guardians and the physician. All consent givers will be informed of their right to bring a lay representative and this is stated in the written information material. If any of the individuals giving the informed consent are not comfortable with the national language, an interpreter fluent in their native language must be provided. The primary investigator will be the direct contact person for the patients and their parents in case of enquiries regarding the project and relevant contact information is stated in the informed consent form.

The PI or the data manager at the participating site will hereafter enter the required data in the online platform used in ALLTogether and randomization will then immediately take place.

Data collection will commence at the time of study inclusion, where diagnostic and demographic data will be registered in the CRF in addition to a baseline physical examination. The physician will confirm in the CRF that the patient meets all inclusion criteria, and that all exclusion criteria are absent.

All adverse event data will be continuously registered in the CRF. Furthermore, the CRF is used to keep account of significant changes in therapy due to occurrence of events. At the end of therapy, blood counts and liver parameters as well as drug doses are registered. Patients will be followed for disease status and SMNs until at least 7 years from end of induction therapy.

#### Objection by minors or incapacitated subjects

Minors or otherwise incapacitated patients that refuse study participation will not be included.

#### Benefits and risks assessment

- Participants in both arms of the TEAM study will have a visit frequency as determined by their treating physician based on symptoms, blood counts, and liver parameters. The TEAM protocol does not prescribe more frequent visits.
- For both study arms, blood sampling for monitoring of MTX/6MP metabolite levels may reveal non-adherence to maintenance therapy, and subsequent improved adherence second to this detection may improve cure rates.
- If the patient is randomized to the experimental arm, study participation could potentially be associated with unexpected toxicities, and thus more frequent hospital visits.
- If the patient is randomized to the experimental arm, the reduced 6MP dosis (and thus reduced levels of MeMP) could lead to a lower frequency of hypoglycemia.

At least once every 3 months blood sampling will include thiopurine/MTX metabolites, but only DNA-TG results from patients in the TEAM experimental arm will be revealed to the treating physician, and then only if DNA-TG is > 1500 fmol/μg DNA at two consecutive measurements within a 3-month period (at least 2 weeks apart).

#### Compensation for injury

Compensation for therapy-related injuries or adverse events will be covered according to the local or national regulations and not by the sponsor.

#### Incentives

Enrolled patients will not receive any economic compensation for participation in this study.

### Administrative aspects, monitoring and publication

#### Handling and storage of data and documents

The handling of personal data will comply with the EU Regulation 2016/679/EU of the European Parliament and of the Council of the 27 April 2016 on the protection of natural persons with regard to the processing of personal data and on the free movement of such data - General Data Protection Regulation (“GDPR”). The Sponsor and Coordinating Investigator will have access to the final trial dataset.

#### Monitoring and quality assurance

As an ALLTogether1 sub-protocol, TEAM will be monitored as specified in the ALLTogether1 Monitoring Plan.

The NPIs are responsible for ensuring that each participating center will have the expertise and procedures to meet any emergency occurring during the study.

The trial will be conducted in accordance with the protocol, legal requirements, and generally accepted quality assurance and quality control procedures (GCP guidelines). To obtain the necessary documentation and monitoring, each NPI will establish an agreement with a national independent GCP unit to perform the monitoring throughout the entire TEAM study.

#### Amendments

Any amendments to this protocol will first be approved by the ALLTogether1 steering committee and the DSMB, and then submitted to the accredited regulatory authorities before being activated.

#### Annual progress report

The annual progress report of the TEAM sub-protocol will be part of the annual ALLTogether1 progress report submitted to the DSMB and national competent authorities.

#### Temporary halt and (prematurely) end of study report

The sponsor will notify the accredited regulatory authorities of the end of the study within a period of 90 days. The end of the study is defined as the last patient’s last follow-up visit or 5 years from that patient’s end of induction therapy, whichever occurs first. The sponsor will notify the accredited regulatory authorities immediately of a temporary halt of the study, including the reason of such an action. In case the study is ended prematurely, the sponsor will notify the accredited regulatory authorities within 15 days, including the reasons for the premature termination. Within 6 months after the end of the study, the investigator/sponsor will submit a final study report with the results of the study, including any publications/abstracts of the study, to the accredited regulatory authorities.

#### Public disclosure and publication policy

This trial will be registered at EudraCT before any patients will be included. All publications will refer to this registration number. All publications from the ALLTogether1 consortium will comply with the guidelines provided by ICMJA (International Committee of Medical Journal Editors) concerning the definition of authorship and ethical rules regarding scientific publications.

The full TEAM protocol is made public through open access publication. Upon study termination, deidentified participant-level data and statistical code will be made available and shared to the extent possible based on the ALLTogether data sharing policy, which is currently under review and will be published on ClinicalTrials.gov before any data are published.

## Discussion

TEAM is a novel approach for maintenance therapy, that is based on a line of supportive studies relating to anticipated toxicity [25, 32], a large observational study on the association between DNA-TG and relapse risk [20], and a pilot study on feasibility and efficacy (with respect to DNA-TG) of the TEAM drug combination [23].

However, only this large randomized trial can determine the efficacy with respect to reduction of relapse risk, and toxicity of the drug combination. Both SOS/NRH and SMN are potential risk factors. The risk of SOS and NRH is anticipated to be low, due to the low dose of 6TG used [32]. Furthermore, SOS has not been associated with DNA-TG [25]. SMN has been associated with higher doses and longer duration of 6MP therapy in previous retrospective studies [36]. An IPD meta-analysis including 1910 patients, of which 14 developed a SMN, did not find an association between DNA-TG and SMN, although larger studies confirming this are warranted [22].

In addition, the risk of infections and osteonecrosis has been considered. However, DNA-TG is not associated with degree of neutropenia [20]; and although most osteonecrosis occur during MT, MTX and 6MP metabolites have not been associated with this complication [37].

The monitoring of MTX and thiopurine metabolites, with reporting back to clinicians on low measures indicating potential non-adherence, may increase treatment adherence in both treatment arms.

We aim to show an improvement in the 5-year DFS from 84 to 91% for BCP-patients (HR 0.54) and 81 to 87% (HR 0.61) for T-cell ALL compared to standard maintenance, through a 50% reduction in relapse rate. The randomization of 778 patients yields a 80% power and a 2-sided 5% alpha to assess this improvement.

## Supplementary Information

**Additional file 1. **List of study sites and Ethical Committees that approved the study.

**Additional file 2. **Lansky/Karnofsky performance scale.

**Additional file 3.** Potential Hy's Law cases.

**Additional file 4. **Study information sheet.

**Additional file 5. **Consent form.

**Additional file 6. **Supplementary Table 1.

## Data Availability

Upon study termination, deidentified participant-level data and statistical code will be made available and shared to the extent possible based on the ALLTogether data sharing statement. Requests for deidentified participant-level data will be evaluated by the ALLTogether Trial Steering Committee.

## Abbreviations

AE: Adverse event
AESI: Adverse event of special interest
ALT: Alanine aminotransferase
ANC: Absolute neutrophil count
AR: Adverse reaction
BCP: B-cell precursor
BSA: Body surface area
CRF: Case report form
CTU: Clinical trial unit
DCR1: Death in first complete remission
DFS: Disease-free survival
DSMB: Data safety monitoring board
DSUR: Developmental safety update report
EFS: Event-free survival
Ery: Erythrocyte
EudraCT: European Union Drug Regulating Authorities Clinical Trials Database
GCP: Good clinical practice
InO: Inotuzumab
IR: Intermediate risk
IRB/EC: Institutional review board/independent ethics committee
IT: Intrathecal therapy
ITT: Intrathecal triple therapy
IVIG: Intravenous Immunoglobulin treatment
MeMP: Methylated 6-mercaptopurine metabolites
6MP: 6-mercaptopurine
6TG: 6-thioguanine
MRD: Measurable residual disease
MTX: Methotrexate
MTXpg: Methotrexate polyglutamates
NPI: National principal investigator
NRH: Nodular regenerative hyperplasia
NUDT15: Nudix hydrolase 15
OS: Overall survival
PI: Principal investigator
R: Randomization
SAE: Serious adverse event
SM: Standard maintenance
SMN: Second malignant neoplasm
SOS: Sinusoidal obstruction syndrome
SmPC: Summary of product characteristics
SUSAR: Suspected unexpected serious adverse reaction
TEAM: Thiopurine enhanced ALL maintenance therapy
TGN: Thioguanine nucleotides
TPMT: Thiopurine methyltransferase
UK: United Kingdom
UNL: Upper normal limit
VOD: Veno-occlusive disease
WBC: White blood cell count
