# Supplementary material for: Thiopurine Enhanced ALL Maintenance (TEAM): study protocol for a randomized study to evaluate the improvement in disease-free survival by adding very low dose 6-thioguanine to 6-mercaptopurine/methotrexate-based maintenance therapy in pediatric and adult patients (0–45 years) with newly diagnosed B-cell precursor or T-cell acute lymphoblastic leukemia treated according to the intermediate risk-high group of the ALLTogether1 protocol
Source: BMC Cancer. 2022 May 2;22:483. doi: 10.1186/s12885-022-09522-3 (PMC9063225; doi:10.1186/s12885-022-09522-3)
Supplement: Supplementary file 1 — Additional file 1. List of study sites and Ethical Committees that approved the study. [file 12885_2022_9522_MOESM1_ESM.pdf]

Appendix 1

List of study sites

Country	Children/Adult	Center name
Belgium	Children	University Hospital Leuven
		University Hospital Ghent
		Cliniques Universitaires Saint-Luc (CUSL)
		University Hospital Brussels
		University Hospital Antwerpen
		Hôpital Universitaire des Enfants Reine Fabiola (HUDERF)
		CHU Liège
		CHC MontLégia
Denmark	Children	University Hospital Rigshospitalet
		Aalborg University Hospital
		H. C. Andersen Børnehospital, Odense Universitetshospital
		Aarhus University Hospital
	Adult	University Hospital Rigshospitalet
		Aarhus University Hospital
Estonia	Children	Tallinn Children´s Hospital
		Tartu University Hospital
	Adult	Tartu University Hospital
		North Estonia Medical Centre
Finland	Children	Helsinki University Hospital
		Kuopio University Hospital
		Tampere University Hospital
		Turku University Hospital
		Oulu University Hospital
	Adult	Helsinki University Hospital
		Kuopio University Hospital
		Tampere University Hospital
		Turku University Hospital
		Oulu University Hospital
Germany	Children	Evangelisches Krankenhaus Bielefeld
		Universitätsklinikum Bonn
		Klinikum Bremen Mitte
		Universitätsklinikum Hamburg-Eppendorf
		HELIOS Klinikum Krefeld
		Universitätsmedizin Mainz
Ireland	Children	Our Lady's Children's Hospital
	Adult	St James Hospital, Dublin
		Haematology Department, St. Vincent's University Hospital

		Haematology Department, Tallaght University Hospital
		Haematology Department, Beaumont Hospital
		Haematology Department, Mater Misericordiae University Hospital
		Clinical Trials Facility, Cork University Hospital, Cork, Ireland
		Clinical Trials Facility, Galway University Hospital
Iceland	Children	Barnaspítali Hringsins Children´s Hospital
	Adult	Landspitali University Hospital
Lithuania	Children	Children's Hospital, Affiliate of Vilnius University Hospital Santaros Klinikos
	Adult	Vilnius University Hospital Santaros Klinikos
Netherlands	Children	Princess Máxima Center for pediatric oncology
Norway	Children	Oslo University Hospital
		Haukeland Hospital
		St. Olavs University Hospital
		University Hospital of North Norway
	Adult	Oslo University Hospital, Rikshospitalet
		Haukeland University Hospital
		St Olavs Hospital
		University Hospital North Norway
		Stavanger University Hospital
Portugal	Children	Instituto Português de Oncologia de Lisboa Francisco Gentil, EPE
		Instituto Português de Oncologia do Porto Francisco Gentil, EPE
		Centro Hospitalar e Universitário de Coimbra, EPE - Hospital Pediátrico de Coimbra
Sweden	Children	Karolinska University Hospital
		Uppsala University Hospital
		Sahlgrenska University Hospital
		Skane University Hospital
		Linköping University Hospital
		University Hospital of Umeå
	Adults	Karolinska University Hospital
		Uppsala University Hospital
		Sahlgrenska University Hospital
		Skane University Hospital
		Linköping University Hospital
		University Hospital of Umeå
		University Hospital of Örebro

List of ethical committees that approved the study

Country	Ethical Committee (local language)	Ethical Committee (English translation)
Belgium	Commissie voor Medische Ethiek	Committee on Medical Ethics
Denmark	National Videnskabsetisk Komite	National Committee on Health Research Ethics
Estonia	Tervise Arengu Instituut	Research Ethics Committee of the Naitonal Institute for Health Development
Finland	Helsingin ja Uudenmaan Sairaanhoitopiiri Eettinen toimikunta	Research Ethics Committee of the Hospital Disctrics of Helsinki and Uusimaa
Germany	Ethik-kommission der Ärztekammer Hamburg	Ethics Committee of the Hamburg Medical Association
Iceland	Vísindasiðanefnd	Scientific Ethics Committee
Lithuania	Lietuvos Bioetikos Komitetas	Lithuanian Bioethics Committee
Netherlands	Medisch Ethische Toetsingscommissie	Medical Ethical Review Committee
Norway	Regionale Komiteer for Medisinsk og Helsefaglig Forskningsetikk REK sør-øst	Regional Committee for Medical and Health Research Ethics South-East Region
Sweden	Etikprövningsmyndigheten	Swedish Ethical Review Authority

April 2022: Ethical approval is pending in France, Ireland, and Portugal. The study will only begin inclusion of patients from these countries, when ethical and regulatory approval has been received.
