# Supplementary material for: Thiopurine Enhanced ALL Maintenance (TEAM): study protocol for a randomized study to evaluate the improvement in disease-free survival by adding very low dose 6-thioguanine to 6-mercaptopurine/methotrexate-based maintenance therapy in pediatric and adult patients (0–45 years) with newly diagnosed B-cell precursor or T-cell acute lymphoblastic leukemia treated according to the intermediate risk-high group of the ALLTogether1 protocol
Source: BMC Cancer. 2022 May 2;22:483. doi: 10.1186/s12885-022-09522-3 (PMC9063225; doi:10.1186/s12885-022-09522-3)
Supplement: Supplementary file 2 — Additional file 2. Lansky/Karnofsky performance scale. [file 12885_2022_9522_MOESM2_ESM.docx]

## Appendix 2

## Lansky/Karnofsky performance scale

| **Karnofsky** | | **Lansky** | |
| --- | --- | --- | --- |
| Score | Description | Score | Description |
| 100 | Normal, no complaints, no evidence of disease | 100 | Fully active, normal. |
| 90 | Able to carry on normal activity, minor signs or symptoms of disease. | 90 | Minor restrictions in physically strenuous activity. |
| 80 | Normal activity with effort; some signs or symptoms of disease. | 80 | Active, but tires more quickly |
| 70 | Cares for self, unable to carry on normal activity or do active work. | 70 | Both greater restriction of and less time spent in play activity. |
| 60 | Required occasional assistance, but is able to care for most of his/her needs. | 60 | Up and around, but minimal active play; keeps busy with quieter activities. |
| 50 | Requires considerable assistance and frequent medical care. | 50 | Gets dressed, but lies around much of the day; no active play, able to participate in all quiet play and activities. |
| 40 | Disabled, requires special care and assistance. | 40 | Mostly in bed; participates in quiet activities. |
| 30 | Severely disabled, hospitalization indicated. Death not imminent. | 30 | In bed; needs assistance even for quiet play. |
| 20 | Very sick, hospitalization indicated. Death not imminent. | 20 | Often sleeping; play entirely limited to very passive activities. |
| 10 | Moribund, fatal processes progressing rapidly. | 10 | No play; does not get out of bed. |
