# Supplementary material for: Thiopurine Enhanced ALL Maintenance (TEAM): study protocol for a randomized study to evaluate the improvement in disease-free survival by adding very low dose 6-thioguanine to 6-mercaptopurine/methotrexate-based maintenance therapy in pediatric and adult patients (0–45 years) with newly diagnosed B-cell precursor or T-cell acute lymphoblastic leukemia treated according to the intermediate risk-high group of the ALLTogether1 protocol
Source: BMC Cancer. 2022 May 2;22:483. doi: 10.1186/s12885-022-09522-3 (PMC9063225; doi:10.1186/s12885-022-09522-3)
Supplement: Supplementary file 3 — Additional file 3. Potential Hy's Law cases. [file 12885_2022_9522_MOESM3_ESM.docx]

## Appendix 3

## Potential Hy’s Law cases

Abnormal values in aspartate transaminase (AST) and/or alanine transaminase (ALT) concurrent with abnormal elevations in total bilirubin that meet the criteria outlined below in the absence of other causes of liver injury are considered potential cases of drug-induced liver injury (potential Hy’s Law cases) and should always be considered important medical events.

The threshold of laboratory abnormalities for a potential case of drug-induced liver injury depends on the patient’s individual baseline values and underlying conditions. Patients who present with the following laboratory abnormalities **should be evaluated further to definitively determine the etiology of the abnormal laboratory values:**

- - - - - Patients with AST *or* ALT *and* total bilirubin baseline values within the normal range who subsequently present with AST or ALT ≥3 times the upper limit of normal (X UNL) concurrent with a total bilirubin ≥2 X UNL, with no evidence of hemolysis or cholestasis and an alkaline phosphatase ≥2 X UNL or not available.
        - For patients with pre-existing ALT *or* AST *or* total bilirubin values above the UNL, the following threshold values are used in the definition mentioned above:
- Pre-existing AST or ALT baseline values above the UNL: AST *or* ALT ≥2 times the baseline values, *and* ≥3 X UNL *or* ≥8 X UNL (whichever is smaller).
- Pre-existing values of total bilirubin above the UNL: Total bilirubin level increased from baseline value by an amount of at least 1 X UNL *or* if the value reaches ≥3 X UNL (whichever is smaller).

The patient should be evaluated as soon as possible, at a center with sufficient resources to carry out and assess all investigations, preferably within 48 hours from awareness of the abnormal results. This evaluation should include laboratory tests, detailed history and physical assessment. In addition to repeating measurements of AST and ALT, laboratory tests should include albumin, creatine kinase, total bilirubin, direct and indirect bilirubin, gamma-glutamyl transferase, prothrombin time (PT), international normalized ratio (INR), and alkaline phosphatase. A detailed history, including relevant information, such as review of ethanol, acetaminophen, recreational drug and supplement consumption, family history, occupational exposure, travel history, history of contact with a jaundiced patient, surgery, blood transfusion, history of liver or allergic disease, and work exposure, should be collected. Further testing for acute hepatitis A, B, or C infection and liver imaging (eg, biliary tract) may be warranted. Cases confirmed on repeat testing as meeting the laboratory criteria defined above, with no other cause for liver function test abnormalities identified at the time should be considered potential Hy’s Law cases irrespective of availability of all the results of the investigations performed to determine etiology of the abnormal liver function test. **Such potential Hy’s Law cases should be reported as SAEs within 24 hours after obtaining knowledge of the event.**
