# Supplementary material for: Thiopurine Enhanced ALL Maintenance (TEAM): study protocol for a randomized study to evaluate the improvement in disease-free survival by adding very low dose 6-thioguanine to 6-mercaptopurine/methotrexate-based maintenance therapy in pediatric and adult patients (0–45 years) with newly diagnosed B-cell precursor or T-cell acute lymphoblastic leukemia treated according to the intermediate risk-high group of the ALLTogether1 protocol
Source: BMC Cancer. 2022 May 2;22:483. doi: 10.1186/s12885-022-09522-3 (PMC9063225; doi:10.1186/s12885-022-09522-3)
Supplement: Supplementary file 4 — Additional file 4. Study information sheet. [file 12885_2022_9522_MOESM4_ESM.pdf]

INSERT HOSPITAL LOGO HERE

INFORMATION SHEET FOR PARENTS

ALLTogether1

TEAM sub-protocol

A trial for children and young adults with acute lymphoblastic leukaemia (ALL)

Randomisation 3 (R3) Intermediate Risk High patients

INFORMATION SHEET (ACUTE LYMPHOBLASTIC LEUKAEMIA)

INTERMEDIATE RISK HIGH PATIENTS ELIGIBLE FOR TEAM

Official title: *A randomised study to evaluate the efficacy of the TEAM strategy for maintenance therapy in paediatric patients and adults (1–45 years) with newly diagnosed B-cell precursor or T-cell ALL treated according to the intermediate risk high group of the ALLTogether1 protocol.*

We would like to invite your child to take part in Randomisation 3 (R3 for short) of a clinical trial run by the Karolinska University Hospital, Sweden, called ALLTogether1. Before you decide whether to let your child take part in the trial we would like you to understand why the trial is being done and what it would involve.

The first information sheet that you received at the start of your child's treatment explained the standard treatment for acute lymphoblastic leukaemia (ALL). This information sheet explains what we are trying to find out in the intermediate risk high arm (that includes R3) of the trial and what treatment will be given to your child if you take part in R3.

You have previously accepted participation for your child in the ALLTogether1 study and to register data about your child's disease, treatment and treatment results in a research database.

Your child is now invited to participate in one of the sub-protocols of the clinical research program, based on the results from the initial treatment phase. This information sheet and the attached consent form contains information to help you make an informed decision whether to let your child participate in the sub-protocol or not. Please read this document carefully. Designated staff (doctors and nurses) are there to answer any questions you may have regarding the study and your child's participation. Take as much time as you need to make your decision. Taking part in this study is voluntary. Deciding not to take part or deciding to leave the study will not affect your child's current or future health care.

Background and purpose

Your child has been diagnosed with acute lymphoblastic leukaemia (ALL). You received information about this disease at the start of the treatment. Patients are divided into different risk-groups depending on the treatment response and some clinical and laboratory features of the disease. This is done to determine how intensive the continued treatment should be. Patients are labelled as Standard, Intermediate (low or high) or High risk and are treated with risk-adapted intensity

accordingly. The prognosis for ALL has improved markedly over time due to better diagnosis, risk categorisation and more effective therapy.

Your child has been assigned to the Intermediate high-risk (IR-high) group. Patients in the IR-high group are identified as having a less favourable prognosis than the Standard and Intermediate Low risk groups, but a more favourable prognosis than the High risk patients. The majority of all relapses in childhood ALL occur in the IR-high group. Following a relapse, 40 % of children can be successfully treated again, and for adults the corresponding figure is less than 20 %, so preventing relapses is very important. New treatment options that improves the antileukaemic efficacy and have an improved safety profile are urgently needed.

Which medicine is being studied?

We are studying a novel medical strategy:
Oral maintenance therapy with the drugs methotrexate and 6-mercaptopurine is an important part of the antileukaemic therapy and is administered until 2.0 years from obtained remission (= control of the leukaemia). Most relapses will occur during or after maintenance therapy. During maintenance therapy 6-mercaptopurine metabolites are built into the leukaemic cells' DNA (=DNA-TG), and high DNA-TG concentration is strongly associated with a reduced risk of relapse. We have demonstrated that DNA-TG levels can be markedely increased by adding to the maintenance therapy the drug 6-thioguanine, which is related to 6-mercaptopurine. In the TEAM study (TEAM = Thiopurine Enhanced ALL Maintenance) patients will be offered this drug combination (methotrexate, 6-mercaptopurine, 6-thioguanine). All three drugs are approved for the treatment of ALL in children. In this study, the TEAM treatment strategy is tested as an alternative to the conventional maintenance therapy of newly diagnosed ALL patient with only methotrexate and 6-mercaptopurine. The aim of the study is to reduce the risk of relapse. The TEAM strategy has been tested in 33 children and young adults with newly diagnosed ALL. The TEAM strategy was well tolerated and able to markedly increase DNA-TG compared to maintenance with only methotrexate and mercaptopurine.

Patients will be randomly assigned by chance (like the flip of a coin) to one of two treatment arms. The randomisation procedure means that everyone has an equal chance of getting any of the three treatment arms:

- **Standard arm -** patients receive conventional maintenance therapy with oral methotrexate and 6-mercaptopurine. In addition they will receive vincristine/dexamethasone pulses at 4 weeks intervals throughout maintenance therapy.
- **Experimental arm TEAM -** patients receive maintenance therapy with oral 6-thioguanine in addition to oral methotrexate and 6-mercaptopurine. The *starting* dose of 6-mercaptopurine will be reduced to make the combination with 6-thioguanine more tolerable and to reduce risk of side effects often seen with 6-mercaptopurine, including liver toxicity and fatigue. All patients in the TEAM arm will in addition to methotrexate and 6-marcaptopurine receive vincristine/dexamethasone pulses at 4 weeks intervals throughout maintenance therapy.

In both study arms the drug doses during maintenance therapy will be adjusted to achieve a preset degree of bone-marrow suppression with a somewhat reduced neutrophil count (white blood cells that combat infections).

If you decide to not take part in the randomisation your child will be treated according to the current standard of care, which is maintenance therapy with oral methotrexate and 6-mercaptopurine. Your doctor will tell you more about the treatment.

Length of study and number of patients who will take part in this study

It is estimated that 878 patients will join TEAM study across several European countries.

Patients will be included in the TEAM study at the start of maintenance, and they will stay on the TEAM study until the end of maintenance therapy, i.e. for a total of approximately 71 weeks. Each patient in the study will be followed for a minimum of five years from inclusion in the randomisation. It is likely that the follow-up for medical reasons will be much longer.

What happens during the study?

Patients in the Intermediate risk-high group will reach the start of maintenance therapy approximately 37 weeks from diagnosis, which is the timepoint for randomisation.

Screening

After you have given written informed consent, your child will first undergo tests to check whether he or she can participate in the randomisation. This is called screening. You are familiar with these tests from the first months of ALL treatment.

Extra tests during the screening are an echocardiogram (ultrasound of the heart), a pregnancy test in patients with child bearing potential, and chemistry/immunoglobulin level (1–2 ml blood). Once these tests have been perfomed, your doctor will assess whether your child can participate in the TEAM sub-protocol.

Treatment

The patients will be randomised to receive

- standard maintenance therapy (**standard arm**); or
- 6-thioguanine added to standard maintenance therapy (**experimental arm TEAM**).

Patients randomised to **standard maintenance therapy** will receive at the start of maintenance therapy:

- 6-mercaptopurine, initial dose 75 mg/m^2/day orally once daily, later adjusted according to blood counts and continued until the end of therapy.
- Methotrexate, initial dose 20 mg/m^2/week orally once weekly, later adjusted according to blood counts and continued until the end of therapy.
- Intrathecal therapy at the start of maintenance therapy and 12-weekly thereafter
- Dexamethasone 6 mg/m^2/day orally, for 5 days at the start of maintenance and then repeated at 4 weeks intervals throughout maintenance.
- Vincristine 1.5 mg/m^2 intravenously at the start of maintenance and repeated every 4-weeks throughout maintenance.

Patients randomised to receive the **TEAM** drug combination will receive at the start of maintenance therapy:

- 6-mercaptopurine, initial dose 50 mg/m^2/day orally once daily, later adjusted according to blood counts and continued until the end of therapy. **Note**, the starting dose of 6-mercaptopurine has been reduced to make the combination with 6-thioguanine more tolerable and to reduce risk of the side effects often seen with 6-mercaptopurine, including liver toxicity and fatigue.
- Methotrexate, initial dose 20 mg/m^2/week orally once weekly, later adjusted according to blood counts and continued until the end of therapy.

- 6-Thioguanine 2.5 mg/m^2/day as an oral liquid formulation, given once daily. If the patient tolerate the dose of 6-thioguanine, the dose will be increased with 2.5 mg/m^2/day every 14 days until a maximum dose of 12.5 mg/m^2/day, if this is tolerated by the patient.
- Intrathecal therapy at the start of maintenance therapy and every 12 weeks thereafter
- Dexamethasone 6 mg/m^2/day orally, for 5 days at the start of maintenance and then repeated at 4 weeks intervals throughout maintenance.
- Vincristine 1.5 mg/m^2 intravenously at the start of maintenance and repeated every 4 weeks throughout maintenance.

Extra blood samples
In the first three months after screening, 4 x 1–2 ml blood in the standard arm. In the long term follow-up extra blood (2 ml) is taken every 12 weeks until the end of maintenance to test liver and kidney function and immunoglobulin levels. This will be analysed locally.

Every month (or at least every three months) blood samples from all patients will be sent to the **TEAM** study center at the Paediatric Research Laboratory "Bonkolab", University Hospital Rigshospitalet, Copenhagen, Denmark, for measurements of methotrexate, 6-mercaptopurine (and 6-thioguanine in the experimental arm) drug metabolite concentrations. At each timepoint the blood sample will be 1–3 ml depending on the childs age. In case of very low drug concentrations in a patient in any of the study arms, your physician will be informed of this to be able to adjust therapy accordingly. In case of very high DNA-TG in a patient in the experimental **TEAM** arm your physician will be recommended to reduce the dose of thioguanine. After drug concentrations has been measured, surplus blood will be stored for quality control until the end of the study, but no more than 15 years, after when they will be destroyed or returned to the treating centre. The stored material will only be used for studies you have consented to.

The details of the procedures and the treatment will be explained to you by your child's doctor. Data from the procedures, treatment, complications, toxicities and follow-up will be collected and registered in the study database.

Which side effects are expected?

The most important side effects of standard maintenance therapy are:
- Low blood counts. The drug doses treatment will then be adjusted accordingly;
- Side effects concerning the liver, including liver enzyme elevations or more rarely a side-effect called VOD (veno-occlusive disease). The latter can be treated with a medicine called defibrotide, if necessary.

To explore if the addition of thioguanine in the **TEAM** experimental arm increases the risk of toxicities known to be associated with maintenance therapy, we will specifically as part of this sub-protocol register data on second cancer (occurs in 1–2% of ALL patients), bone damage (socalled osteonecrosis), low levels of immunoglobulins (antibodies that protects against infections), hypoglycaemia, and liver toxicity.
Other known side effects of maintenance therapy are listed in Appendix B. Side effects that are not yet known may also occur. You will receive all new information that may influence your child's health.
The maintenance therapy administered to your child in the context of one of the sub-protocols is a treatment phase that is always used to treat patients with ALL. There are potential known side effects associated with the medicines given. Problems or side effects that are not known could also occur.

What are the possible benefits and disadvantages of taking part in this randomisation?

Children and young adults with IR-high ALL have a worse prognosis compared to patients classified to lower risk groups. There is a need for treatment options that improve the efficacy of treatment and decrease toxicity.

Experimental arm – **TEAM**: A previous study has shown that the level of DNA-TG (=the antileukaemic metabolite of both 6-mercaptopurine and 6-thioguanine) is strongly associated to the risk of relapse. Thus, patients with high DNA-TG levels seems to have a lower risk of relapse than patients with low DNA-TG levels. Researchers in the ALLTogether consortium has tested the TEAM treatment strategy in 33 children and young adults with ALL, and shown that it is well tolerated and that is leads to much higher DNA-TG levels than conventional maintenance therapy with only methotrexate and 6-mercaptopurine. An additional advantage is that high DNA-TG can be obtained with lower doses of 6-mercaptopurine. Since 6-mercaptopurine frequently causes liver toxicity with liver enzyme elevations, fatigue and a risk for low blood sugar, TEAM may reduce the risk of these side-effects. For patients in the experimental TEAM arm, your child's physician will also be informed of extremely high DNA-TG levels in order to reduce the 6-thioguanine dose, since we do not know if these very high DNA-TG concentrations could be associated with an increased risk of side effects.

Participants in the study are offered frequent measurements of drug concentrations. Very low concentrations will be reported back to the physician responsible for your child's treatment who can then adjust therapy accordingly. This, may lead to better drug dosing.

Disadvantages: The disadvantages are the possible side effects of Thioguanine and the extra blood tests as described above. There may also be unknown or severe side effects.

There is no guarantee that your child will benefit from the participation in this randomisation, but the specific information obtained as a result of your child's participation will help to increase our general knowledge of the disease and its treatment to the benefit of future patients. The fact that the results are continuously monitored could also lead to safety measures or adapted treatment even during the study.

Confidentiality

All information collected about your child for this trial will be subject to the laws of confidentiality according to GDPR. Further details about this are found in the information sheet concerning participation and registration.
With your permission your child's doctor will notify your child's GP that he/she intends to participate in the trial. We may also share information with other hospitals involved in your child's care.
If you choose to withdraw your child from the trial treatment we would still like to collect relevant information about your child's health, as this will be invaluable to our research. If you have any objection to this please let your child's doctor know.
Under no circumstances will you be identified in any way in any report, presentation or publication arising from this trial.

The data on maintenance therapy drug doses, blood counts and drug concentrations will be stored in a database located on a secured server at the University Hospital Rigshospitalet in Copenhagen, Denmark. The other data collected in the study will be stored in a database located on a secured server in the Netherlands.

Some information about the TEAM study (mostly information about side-effects of Thioguanine) may be transferred to the Nova Laboratories Ltd. Nova Laboratories Ltd is supporting the study by providing the study medication (liquid formulation of thioguanine) and has a legitimate cause to find out about such side-effects to develop the medicine. However, information that may be transferred from the study center will not include any information about the identity of your child or your child's personal data.

Data Protection

All processing of your child's personal data will be carried out in strict compliance with applicable data protection (Insert name of applicable national legislation), the EU Regulation (EU) 2016/679 of the European Parliament and of the Council of the 27 April 2016 on the protection of natural persons with regard to the processing of personal data and on the free movement of such data (General Data Protection Regulation, GDPR).

The University Hospital Rigshospitalet, Copenhagen, Denmark, is the data processor in the TEAM study for all the pharmacological data, and is responsible for the processing of your data. Karolinska University Hospital is the data controller in this study with respect to your child's other disease and treatment data and responsible for the processing of your child's personal data. We are obliged to keep the study data for 25 years. You give permission for this when your child participates in this study. If you do not agree, your child may not participate in this study.

In addition to your child's treating physician, the following individuals will also have access to your child's data:

- the study team,
- the monitor/auditor,
- employees of or people appointed by Nova Laboratories Ltd to check and guide the study,
- the medical ethics committee, and the safety committee that monitors the study,
- the Health Care Inspectorate and inspectors from foreign governments (such as the EMA).

Voluntary participation

It is completely voluntary to participate in the study and you can at any time without explaining why, withdraw your consent without this affecting your child's medical care. However, the data already reported will not be deleted in accordance with current rules. Your child do not have to take part in this study to receive treatment or care.

We will tell you about any new information that may affect your child's participation in the study. You can then decide if you want your child to continue in the study and with other study-related activities.

Results

When the trial is complete the results will be published in medical journals and presented at scientific meetings, but no individual patients will be identified. If you would like to obtain a copy of the published results, please ask your doctor or nurse.

Insurance

It is a legal requirement that all patients included in the study are covered by insurance. This is organized {INSERT APPLICABLE NATIONAL SOLUTION FOR INSURANCE COVER}.

The Sponsor of the study, Karolinska University Hospital, Stockholm, Sweden will not be financially responsible for unforeseen side effects of the study.

Would you like to know anything more?

If you have any questions or complaints during the study, you can always contact your child's study doctor.
If you have questions or comments, you can also contact:

- The national study Principal Investigator <add PI name and title>, <add specialization e.g paediatrician-oncologist>, tel. <add phone number>, national principal investigator.
- The study nurses of the <paediatric oncology> department, tel. <add phone number>.
- Or send an e-mail to <add email address>.

If, after careful consideration, you decide that your child will indeed participate in this study, we ask that you sign and date the informed consent form together with the doctor.
Take your time to discuss this information, and do not hesitate to contact the study doctor if you have any questions. If you decide to participate, you will receive a copy of this document after you and the study doctor have both signed for participation.

The international **TEAM** sub-protocol chair:
Kjeld Schmiegelow
Professor in Paediatrics and Paediatric Oncology
University Hospital Rigshospitalet, Copenhagen, Denmark

The ALLTogether1 protocol Chief Investigator:
The Chief Investigator who is responsible for the conduct of the ALLTogether1 study is Dr Mats Heyman (Karolinska University Hospital, Sweden).
For questions regarding your personal data contact local Data Protection Officer (addresses and phone numbers of the local DPO/University Hospital and local Data protection agency).

Appendices
Appendix A treatment schedule and pharmacology
Appendix B Side effects
Appendix C General information<add general information leaflet if available and adjust to country specific situation.> <example NL (remove when creating country specific version): General leaflet on medical-scientific research and leaflet from the VOKK in the diary>

Appendix A-2 Treatment schedule **TEAM**

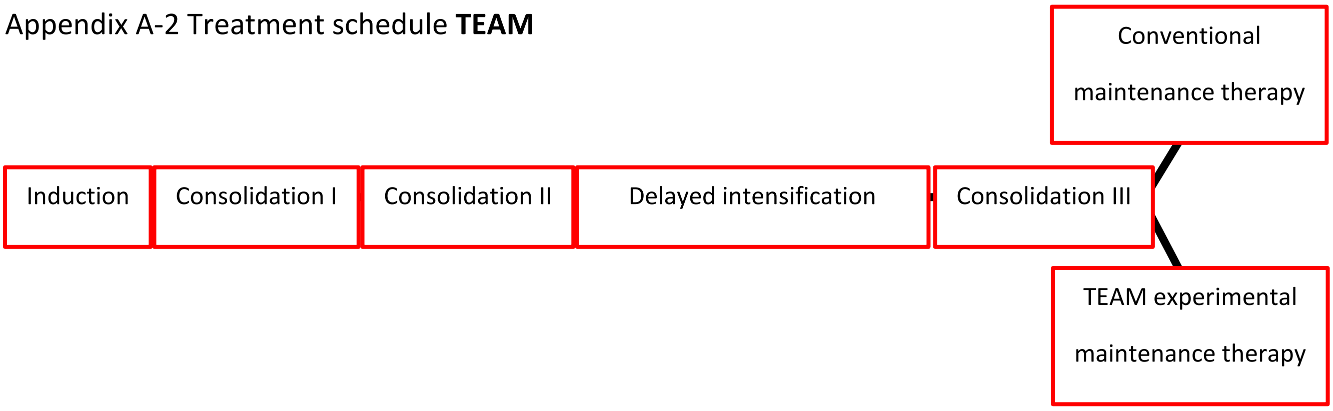

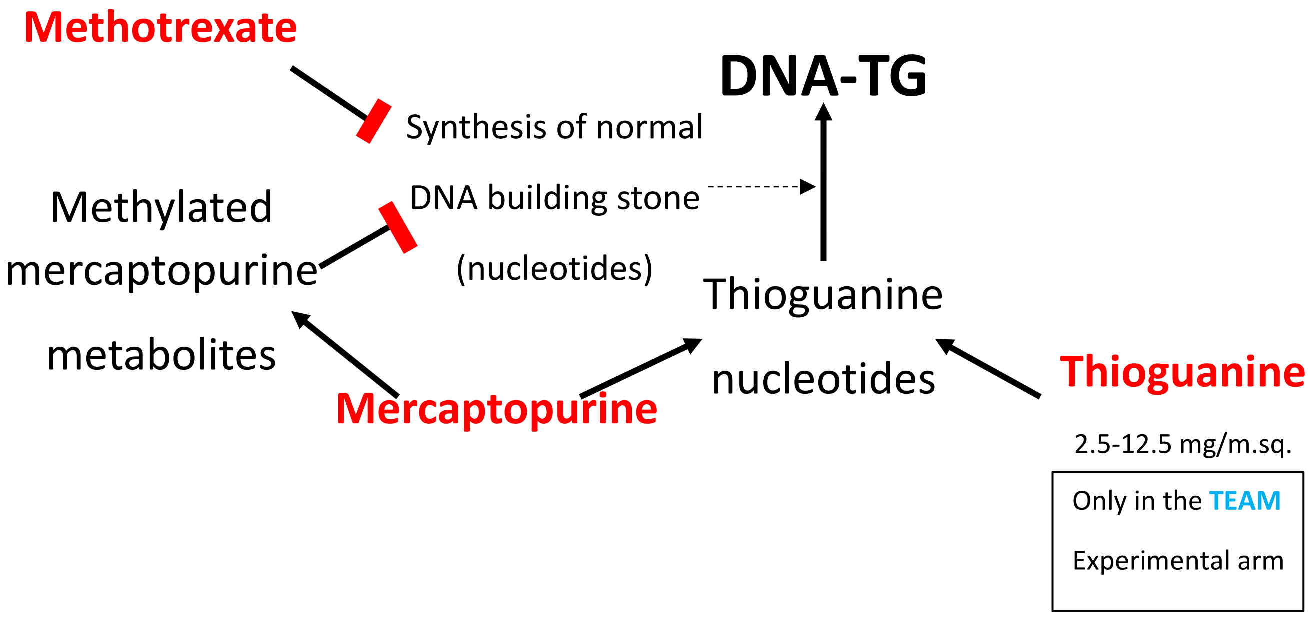

The antileukaemic effect of maintenance therapy is primarily mediated by the accumulation of thioguanine nucleotides in DNA (= DNA-TG). The thioguanine nucleotides looks very much like the normal DNA building stones (so called nucelotides), but they can cause DNA damage and death of the leukaemic cells. During maintenance therapy the DNA-TG level varies 10-fold between patients. Higher DNA-TG levels seems to be associated with reduced risk of relapse of the leukaemia. The thioguanine nucleotides are build into DNA in competition with the normal DNA building stones (nucleotides). Both methotrexate and some of the mercaptopurine metabolites can reduce the production of the normal DNA building stones, and thus enhance the incorporation of thioguanine nucleotides into DNA. When thioguanine is added to the conventional maintenance therapy, it is more easily converted into thioguanine nucleotides (thicker arrow) than is the case for mercaptopurine. The co-administration of thioguanine to a backbone of methotrexate and mercaptopurine will reduce the production of the normal DNA building stones, and thus increase the incorporation of thioguanine nucleotides into DNA leading to significantly higher DNA-TG levels and potentially a reduced risk of relapse.

Appendix B-1 Side effects TEAM

The table below shows you which side effects can be observed with maintenance therapy. A distinction has been made between common side effects that were observed in more than 10% of the patients and side effects that occurred in less than 10% of the patients. Other side effects can also occur and side effects that were not previously observed with use of TEAM can be reported.

Please contact your doctor immediately, if you think that your child has experienced a severe side effect?

Examples of a severe side effect include:

- A severe liver condition called VOD (veno-occlusive disease) or NRH (Nodular Regenerative Hyperplasia). VOD is a condition that is often associated with rapid weight gain, pain on the right side of the abdomen, enlargement of the liver, fluid retention, swollen abdomen, increase in bilirubin and jaundice. NRH is a chronic liver toxicity, with a risk of increased pressure in the blood vessels of the liver. SOS or NRH has been described in ALL patients receiving doses of 6-thioguanine that are much higher than the doses used in TEAM, typically 40 mg/m^2 or more. In the TEAM experimental arm, these side effects are expected to occur in less than 1 in 20 patients. Should they occur your child will be taken of the TEAM experimental arm and instead receive conventional maintenance therapy with methotrexate and 6-mercaptopurine only.
- Very low blood counts, such as white blood cells (neutrophils) and blood platelets can cause infections and bleeding, and is known side effects of maintenance therapy and are nearly always transient. These side effects occur in more than 1 in 10 patients.

**Organ and type of side effect**	**Very Common (10% or more)**	**Common (5%–10%)**	**Rare (<5%)**
Infections	Infections (various types)		
Low blood counts	Fever in aplasia<br>Low number of white blood cells (neutrophils, leucocytes, and / or lymfocytes)<br>Low number of blood platelets<br>Anaemia	Pancytopenia	
Metabolism and diet	Loss of appetite		
Gastrointestinal abnormalities		Abdominal pain, vomiting, nausea	
Liver abnormalities	Increased liver enzymes		Veno-occlusive disease or nodular regenerative hyperplasia
General condition	Fatigue, fever		
