# Supplementary material for: Thiopurine Enhanced ALL Maintenance (TEAM): study protocol for a randomized study to evaluate the improvement in disease-free survival by adding very low dose 6-thioguanine to 6-mercaptopurine/methotrexate-based maintenance therapy in pediatric and adult patients (0–45 years) with newly diagnosed B-cell precursor or T-cell acute lymphoblastic leukemia treated according to the intermediate risk-high group of the ALLTogether1 protocol
Source: BMC Cancer. 2022 May 2;22:483. doi: 10.1186/s12885-022-09522-3 (PMC9063225; doi:10.1186/s12885-022-09522-3)
Supplement: Supplementary file 5 — Additional file 5. Consent form. [file 12885_2022_9522_MOESM5_ESM.pdf]

ALL Together

INSERT HOSPITAL LOGO HERE

Site Name: <<insert site name or site number>>

Patient Study ID: <<insert patient study number and patient name>>

CONSENT FORM R3 2-ARM RANDOMISATION

Name of Study: ALLTogether1 TEAM Sub-protocol - Randomisation 3 (R3)

Official title: A randomized study to evaluate the efficacy of the TEAM strategy for maintenance therapy in pediatric patients and adults (1-45 yrs) with newly diagnosed B- or T-cell precursor ALL treated according to the intermediate risk high group of the ALLTogether-1 protocol.

Name of local Principal Investigator: <<insert name of Principal investigator>>

If applicable, IRAS No.: [insert IRAS number]

Please initial box

1.	I confirm that I have read the information sheet dated <Insert final version and date> for the above study. I have had the opportunity to consider the information, ask questions and have had these answered satisfactorily.	☐
2.	I understand that my participation is voluntary and that I am free to withdraw at any time, without giving any reason and without my medical care or legal rights being affected.	☐
3.	I understand that relevant sections of my medical notes, and data collected during the study, may be looked at by representatives from the study sponsor, Karolinska University Hospital, Sweden and <<insert country-specific agencies including co-ordinating centre>>, local/national <<insert relevant healthcare institution>> and from relevant regulatory authorities.	☐
4.	I agree to my General Practitioner (GP) being informed of my participation in this study.	☐
5.	I give permission for data, tissue and blood samples can be used for research related to the study, and if the data are protected sufficiently by means of a coding system.	☐

6.	**If appropriate and data sharing now or in the future is planned, include the following:**<br><br>I understand that the information collected about me will be used to support other research in the future, and may be shared with other researchers, in which case items that could directly identify me would be removed and a code used to link information.	☐
7.	I agree to take part in the above study.	☐

Name of Parent/Guardian	Date	Signature
______________________	__________	______________________
Name of Parent/Guardian	Date	Signature
______________________	__________	______________________
Name of person taking consent (designated responsible person)	Date	Signature
______________________	__________	______________________

Instructions to sites: When completed: Take 2 copies. Original and 1 copy to be kept in medical notes and investigator site file, and a copy to be given to the parent/guardian.
