# Supplementary material for: Thiopurine Enhanced ALL Maintenance (TEAM): study protocol for a randomized study to evaluate the improvement in disease-free survival by adding very low dose 6-thioguanine to 6-mercaptopurine/methotrexate-based maintenance therapy in pediatric and adult patients (0–45 years) with newly diagnosed B-cell precursor or T-cell acute lymphoblastic leukemia treated according to the intermediate risk-high group of the ALLTogether1 protocol
Source: BMC Cancer. 2022 May 2;22:483. doi: 10.1186/s12885-022-09522-3 (PMC9063225; doi:10.1186/s12885-022-09522-3)
Supplement: Supplementary file 6 — Additional file 6. Supplementary Table 1. [file 12885_2022_9522_MOESM6_ESM.pdf]

Supplementary Table 1.

Cox model for time to relapse for patients treated according to NOPHO ALL2008 fulfilling ALLTogether Intermediate Risk-high criteria

No of patients/samples/relapses	225/2 846/16	
	HRa (95% CI)	p
$_{wm}$DNA-TG	0.61 (0.43–0.87)	0.0068
Age at diagnosis	1.04 (0.93–1.15)	0.51
Female sex	1.07 (0.40–2.89)	0.89
White blood cell count at diagnosis	1.01 (0.96–1.05)	0.78

CI, confidence interval; $_{wm}$DNA-TG, weighted mean of DNA-incorporated thioguanine nucleotide; HRa, adjusted hazard ratio per 100 fmol/µg increase in DNA-TG, per 1-year increase in age, and per $10{\times}10^9$/L increase in white blood cell count at diagnosis.
